# An Integrative Computational Approach for the Prediction of Human-*Plasmodium* Protein-Protein Interactions

**DOI:** 10.1155/2020/2082540

**Published:** 2020-12-19

**Authors:** Kais Ghedira, Yosr Hamdi, Abir El Béji, Houcemeddine Othman

**Affiliations:** ^1^Laboratory of Bioinformatics, Biomathematics and Biostatistics (LR16IPT09), Pasteur Institute of Tunisia, 1002, University of Tunis El Manar, Tunis, Tunisia; ^2^Laboratory of Biomedical Genomics and Oncogenetics, LR16IPT05, Pasteur Institute of Tunis, University of Tunis El Manar, Tunis, Tunisia; ^3^Institut National des Sciences Appliquees et de Technologie, Université Carthage, Tunis, Tunisia; ^4^Sydney Brenner Institute for Molecular Bioscience, Faculty of Health Sciences, University of the Witwatersrand, Johannesburg, South Africa

## Abstract

Host-pathogen molecular cross-talks are critical in determining the pathophysiology of a specific infection. Most of these cross-talks are mediated via protein-protein interactions between the host and the pathogen (HP-PPI). Thus, it is essential to know how some pathogens interact with their hosts to understand the mechanism of infections. Malaria is a life-threatening disease caused by an obligate intracellular parasite belonging to the *Plasmodium* genus, of which *P. falciparum* is the most prevalent. Several previous studies predicted human-plasmodium protein-protein interactions using computational methods have demonstrated their utility, accuracy, and efficiency to identify the interacting partners and therefore complementing experimental efforts to characterize host-pathogen interaction networks. To predict potential putative HP-PPIs, we use an integrative computational approach based on the combination of multiple OMICS-based methods including human red blood cells (RBC) and *Plasmodium falciparum* 3D7 strain expressed proteins, domain-domain based PPI, similarity of gene ontology terms, structure similarity method homology identification, and machine learning prediction. Our results reported a set of 716 protein interactions involving 302 human proteins and 130 Plasmodium proteins. This work provides a list of potential human-*Plasmodium* interacting proteins. These findings will contribute to better understand the mechanisms underlying the molecular determinism of malaria disease and potentially to identify candidate pharmacological targets.

## 1. Introduction

Infectious diseases represent a major public health challenge that results from molecular cross-talks between pathogens and their hosts. These cross-talks are mostly mediated by protein-protein interactions occurring between host and pathogen (HP-PPI). PPI correspond to physical interactions between proteins that represent the key elements of the infection mechanism and play crucial roles in the evolution of the infections, as they may turn the balance in favor of the spread of the pathogen or its clearance. Malaria is an infectious disease caused by one of the five types of the protozoan parasite Plasmodium with *P. falciparum* strain being the most prevalent. The infection is transmitted by an infected mosquito that bites a human, leading to the multiplication of the parasites in the host's liver before infecting and destroying red blood cells. In 2018, WHO estimated 228 million cases of malaria occurring worldwide (95% confidence interval [CI]: 206-258 million) with around 405000 deaths from malaria globally (https://www.who.int/news-room/feature-stories/detail/world-malaria-report-2019), of which 93% of them are recorded in Africa. Thus, identifying the human and Plasmodium proteins involved in the infection can provide insights into the underlying molecular mechanisms of pathogenicity and potentially identify new putative pharmacological targets. Experimental methods that have been used to predict the host-pathogen protein interactions include the yeast two-hybrid (Y2H) system, affinity purification (AP) [1] coupled to mass spectrometry (MS) [2], proximity-dependent labeling coupled to mass spectrometry, chemical crosslinking coupled to mass spectrometry (XL-MS), and protein microarray methods. These later have been extensively used to capture the interactions between host and microbial proteins at different resolution levels [3–6]. Although these techniques are able to successfully identify host-pathogen proteins interactions, their technical challenges [7] and their high cost impedes their application and feasibility [8]. Nowadays, computational methods have shown utility in performing large screening, improving the accuracy and efficiency for identifying protein-protein interactions in combination with experimental data sets [9, 10]. Moreover, these computational tools are contributing in complementing large-scale experimental efforts to characterize host-pathogen interaction networks. Numerous efforts have previously investigated host-pathogen interactions in the context of malaria disease using computational methods [11–19]. Depending on the methods used, the predictions may include large false positive interactions. Here, we present an integrative computational approach for the prediction of host-pathogen protein-protein interactions based on the combination of six distinct approaches including protein sequence homology, domain-domain protein interactions, proteins similarity structure, similar Gene Ontology terms, and the use of human and parasite expression data to predict human-*Plasmodium falciparum* 3D7 interactions combined to machine learning techniques in order to better understand the mechanisms underlying the malaria disease.

## 2. Materials and Methods

### 2.1. Data Integration

In order to decrease false-positive predictions, we have performed an integrative computational approach by integrating five distinct OMICS based approaches and different datasets.

#### 2.1.1. Protein Expression Data

We collected lists of gene names related to mass-spectroscopic proteome analyses of human red blood cells (RBC) from two distinct resources. We have integrated 1,578 human proteins previously reported as expressed in RBC [20]. We also included a recently updated and improved dataset of RBC proteome [21], which reports a nonredundant list of 1,989 gene products. Furthermore, expression profile (peak protein expression stage) and subcellular localization of *Plasmodium falciparum* 3D7 proteins for merozoite, ring, trophozoite, and schizont stages were extracted from PlasmoDB and from other studies published earlier [22, 23]. To recognize putative interactions brought about by membrane proteins of the vacuole, we included parasite proteins that are previously established as parasitophorous vacuole membrane proteins [24]. Moreover, we included parasite proteins reported to be associated with Maurer's cleft specialized secretory compartment [25]. Finally, merozoite surface proteins that are involved in host RBC invasion were also included [26]. In total, we obtained 2,430 nonredundant plasmodium RBC-expressed proteins and 1,889 unique human RBC-expressed proteins.

#### 2.1.2. Host-Pathogen Protein-Protein Interaction Based on Domain-Domain Interactions

It is well established that protein domains are the key mediators of any protein-protein interaction. Exploiting domains as building blocks for PPI prediction have been widely used [27, 28]. Several databases are available to provide open access to domain-domain interaction data. Here, we collected (On February 2020) data from the INstruct database accessible through http://instruct.yulab.org/ [29] and iPfam database, available at http://ipfam.org [30] providing high-quality 3D structurally resolved protein-protein interactions and Pfam domain interactions based on known 3D structures found in the Protein Data Bank, respectively.

#### 2.1.3. Host-Pathogen Protein-Protein Interaction Based on Gene Ontologies

Protein partners from PPIs may participate in related and/or similar biological processes [12]. The gene ontology (GO) project offers a standardized annotation schema for proteins involved in specific biological processes [31]. To identify potential host-pathogen protein interactions based on their involvement in similar and/or related biological processes, Human and *Plasmodium falciparum* 3D7 gene ontologies and annotation information were retrieved (On February 2020) from the Gene Ontology project website http://current.geneontology.org/annotations/index.html.

#### 2.1.4. Host-Pathogen Protein-Protein Interaction Homology Based

The rationale behind the homology-based method is that conserved interactions between a pair of proteins are expected to have interacting homologs in other species. The conserved interaction is called “Interolog.” Considering a template PPI pair (*x*, *y*) in source species, identify the homolog *x*′ in the host and the homolog *y*′ in the pathogen and then conclude that (*x*′, *y*′) pair also forms a PPI [32]. Known host-pathogen protein interactions were retrieved (On February 2020) from Phi-Base database (http://www.phi-base.org/index.jsp) [33], protein-protein interactions derived from Reactome database (http://www.reactome.org) [34], Biogrid: The Biological General Repository for Interaction Datasets (BioGRID: https://thebiogrid.org) [35], and Intact (http://www.ebi.ac.uk/intact) [36]. Homology relationship was determined between protein sequences of *P. falciparum* 3D7 collected from PlasmoDB (https://plasmodb.org/common/downloads/) against human protein sequences. Blastp Best reciprocal hit (BRH) approach with *E*value ≤ 10^−5^ was used for homology investigation [37].

#### 2.1.5. Host-Pathogen Protein-Protein Interaction Structure Based

Multiple studies used a structure similarity-based method and use template PPIs to detect similar interacting pairs within host and pathogen proteins [11]. Such a method starts with a set of host and pathogen proteins, and then sequence matching procedures are used to determine the similarities between the host or pathogen proteins with known structure or known interaction protein partners. Data for structurally known interaction protein partners integrated here were retrieved (On February 2020) from PrePPI (http://bhapp.c2b2.columbia.edu/PrePPI) [38], SNAPPI-DB (http://www.compbio.dundee.ac.uk/SNAPPI/downloads.jsp) [39], SCOP2 (http://scop2.mrc-lmb.cam.ac.uk/), [40], and the database of three-dimensional interacting domains (3did) (https://3did.irbbarcelona.org/) [41].

#### 2.1.6. Data Standardization and Harmonization

Collected data from several databases were structured in different formats with different features. They were parsed to extract relevant information. Accession IDs in UniProt were mapped to HUGO Gene Nomenclature Committee (HGNC) symbols for human and PF IDs for Plasmodium. We also used PlasmoDB gff files to convert PF ID into aliases (example: PF ID = PF3D7_0302600, Alias = PFC0125w). Final results were presented into a tab-separated file format displaying the potential interactions between Human genes (HGNC symbol) and *Plasmodium falciparum* 3D7 genes (PF ID and aliases).

#### 2.1.7. Functional Analysis

Human and Plasmodium proteins involved in predicted interactions using the present computational approaches were subject to gene set enrichment analysis in order to identify significantly enriched pathways. Functional analysis was performed using StringDB [42].

### 2.2. Host-Pathogen Interaction- (HPI-) Prediction Using Machine Learning Protein Sequences Based Approaches

#### 2.2.1. Data Preprocessing

Domain-domain interaction data collected (On February 2020) from the3DID database and other data from [43] were used as positive and negative protein-protein interactions to train the models. For each protein or domain accession, amino-acid sequences were retrieved, and for each pair of interactors, sequences were concatenated. Thereafter, amino-acid sequences have been converted into overlapping 3-mer subsequences, and occurrences of all subsequences were counted with term frequency-inverse document frequency (TF-IDF) vectorizer provided by Scikit-Learn python module. TF-IDF vectorizer is a very common algorithm for text analysis for machine learning, by evaluating how relevant a subsequence is associated to a sequence in a collection of all sequences. This is done by multiplying two metrics to know how many times a 3-mer subsequence appears in the whole sequence and the inverse document frequency of the subsequence across the set of amino-acid sequences. (ngram_range = 2, 2 (bigrams), max features = 2000 (top features ordered by term frequency across the sequence)) [44].

#### 2.2.2. Machine Learning Classifiers

Eight classifiers have been evaluated, namely, the *K*-nearest neighbors (KNN) classifier [44], the logistic regression classifier [45], the decision tree [46], the random forest [47], the adaptative boost (Adaboost) [47] classifier, the voting classifier [48], the Gaussian Naive Bayes classifier [46], and the support vector machine (SVM) [49].

#### 2.2.3. Performance Metrics

For validation, *k*-fold cross-validation is used. It is a powerful preventative measure against overfitting. *K* is fixed at 10, which means that the training dataset is divided into 10 equal parts and the process will run 10 times, each with a different holdout set. This allows us to keep our test set as an unseen dataset for selecting the final tuned model [50]. To evaluate the performances of the studied classifiers, we estimated the five measures below:

- *Precision*. It refers to the percentage of results, which are relevant. It is the ratio of correctly predicted positive observation to the total positive observations
- *Recall*. It refers to the percentage of total relevant results correctly predicted points out of all the data points
- *Accuracy*. It is the number of correctly predicted labels out of all the class labels
- *F1-Score*. It is the weighted average of Precision and Recall. It takes both false positive and false negatives into account
- *AUC*. The area under the curve is the measure of the ability of a classifier to distinguish between classes and is used as a summary of the ROC curve
- *ROC Curve*. It plots true positive rate (sensitivity) on the *y*-axis and false positive rate (specificity) on the *x*-axis

The five performance measures are defined as follows:

(1) $$\begin{array}{c} \text{Precision}=\frac{\text{TP}}{\text{TP}+\text{FP}}, \end{array}$$

(2) $$\begin{array}{c} \text{Accuracy}=\frac{\text{TP}+\text{TN}}{\text{TP}+\text{TN}+\text{FP}+\text{FN}}, \end{array}$$

(3) $$\begin{array}{c} F-\text{score}=2*\text{TPR}*\frac{\text{Precision}}{\text{TPR}+\text{Precision}}, \end{array}$$

(4) $$\begin{array}{c} \text{TPR}=\frac{\text{TP}}{\text{TP}+\text{FN}}, \end{array}$$

(5) $$\begin{array}{c} \text{FPR}=\frac{\text{FP}}{\text{FP}+\text{TN}}, \end{array}$$

where TP, TN, FP, FN, TPR, and FPR represent true positive, true negative, false positive, false negative, true positive rate, and false-positive rate, respectively (see Supplementary File 1 and Supplementary File 2).

## 3. Results

**Figure 1:**
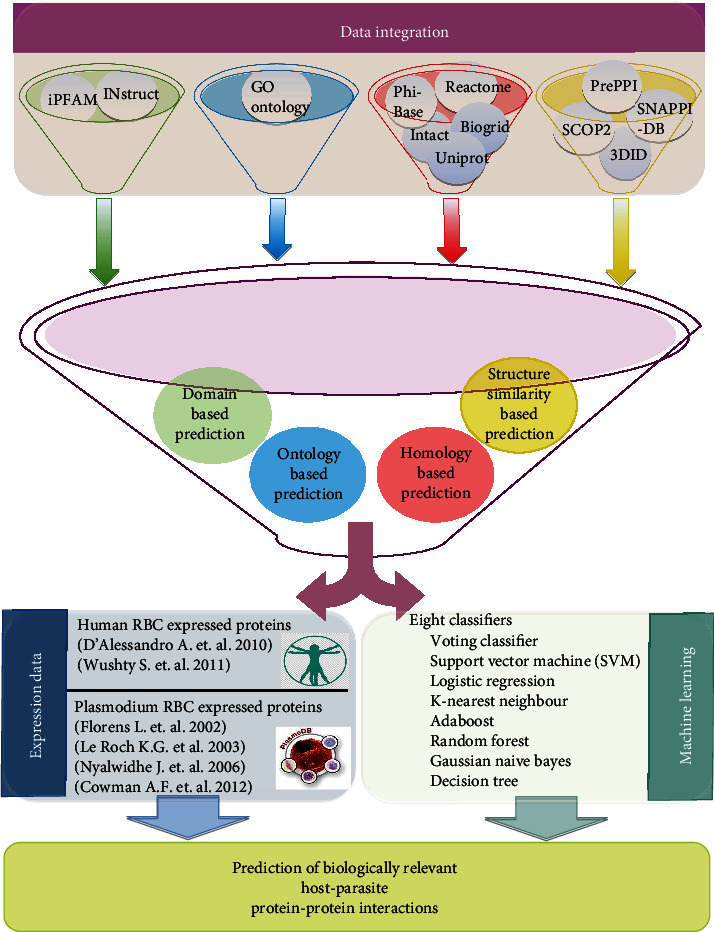
Data integration schema.

In the present study, we integrated data from different sources combining several approaches that have been widely used to predict host-pathogen interacting proteins (Figure 1).

### 3.1. HPI Prediction Based on Machine Learning Approach

Table 1 highlights the performance of each investigated classifier based on calculated metrics. We observed that most of the individual classifiers tend to perform effectively.

**Figure 2:**
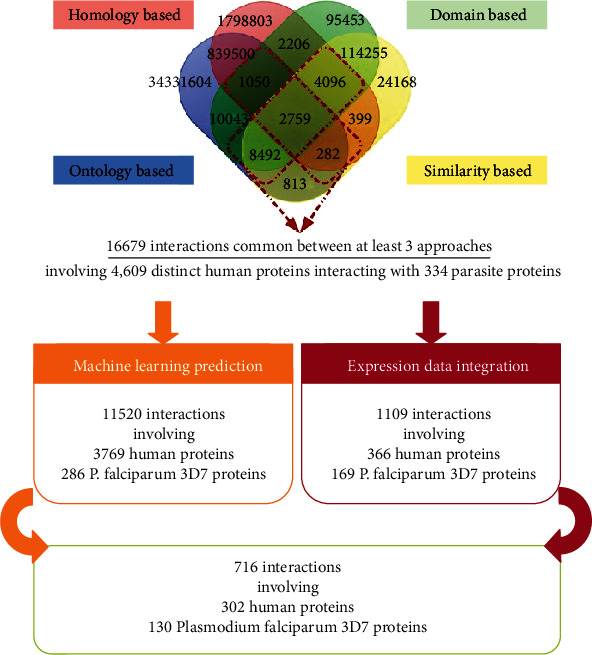
Comparison between the different approaches. The Venn diagram was created using the Venn diagram tool (http://bioinformatics.psb.ugent.be/webtools/Venn/).

Figure 2 highlights the overlap between human-*Plasmodium falciparum* 3D7 parasite interacting proteins after applying the four filters described previously, i.e., domain-domain interactions, protein structure similarity, ontology-based filter, and homology to partners in known PPIs. In order to be less stringent, we selected host-pathogen interactions that were common to at least 3 distinct filters. Our results showed a total of 16,679 (1050 + 4096 + 8492 + 282 + 2759) host-pathogen putative interactions involving 4,609 distinct human proteins and 334 different parasite proteins.

### 3.2. The Integrative Approach

Considering the whole sets of distinct 2,430 plasmodium RBC-expressed proteins and 1,889 human RBC-expressed proteins integrated in the present analysis, the number of possible interactions across the host erythrocyte and the parasite proteins would tremendously be very large. To reduce false-positive predictions, we have combined appropriate approaches as previously detailed, thereby resulting in the prediction of probable host-parasite interactions. A detailed representation of the approach followed is shown in Figure 2.

**Figure 3:**
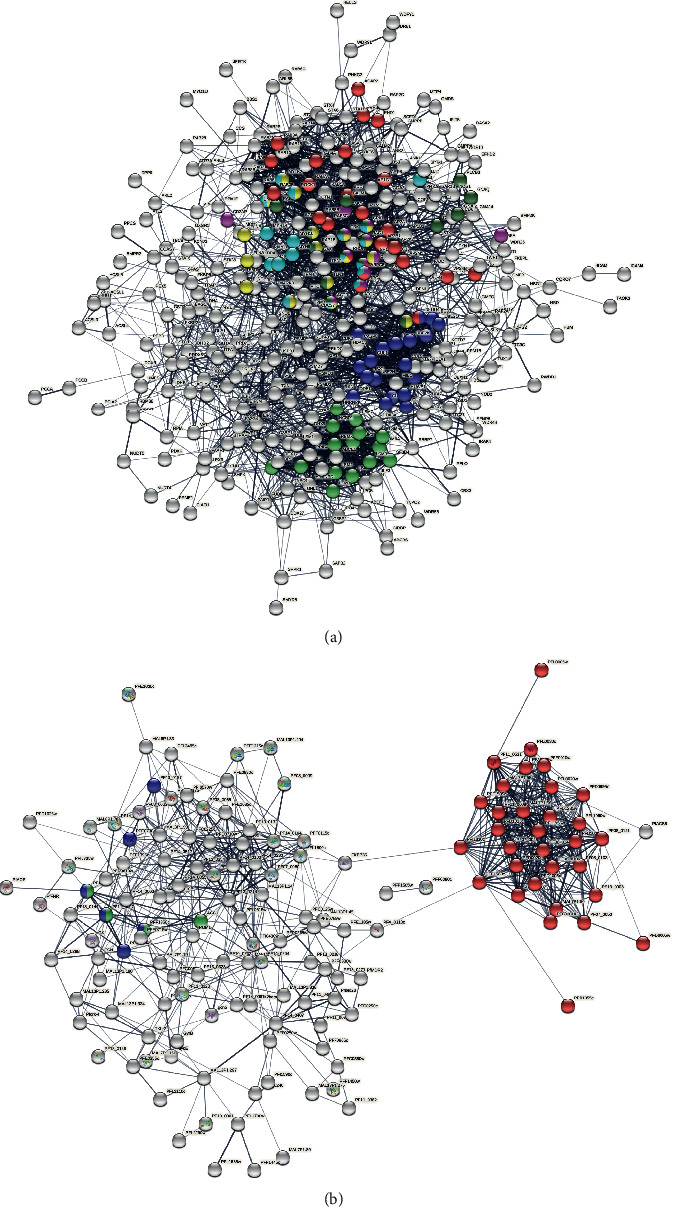
Protein-protein interactions network generated using StringDB. (a) The human interactome involving the 366 RBC-expressed genes identified through the combination of different approaches. Nodes in red color denote proteins involved in endocytosis. Those in blue color denote proteins involved in ubiquitin-mediated proteolysis. Green color corresponds to proteins involved in spliceosome. Yellow nodes denote proteins implicated in focal adhesion. Pink color represents proteins involved in bacterial invasion of epithelial cells, while cyan color highlights proteins related to regulation of actin cytoskeleton. (b) The *Plasmodium falciparum* 3D7 DEG interactome. Nodes in red color denote proteins involved in the malaria pathway. Nodes with blue color denote proteins involved in “Carbon metabolism.” Green color corresponds to proteins involved in the Propanoate metabolism.

Among the 4,609 human proteins involved in the predicted interactions using at least three distinct approaches, only 366 (out of 1,889 human RBC-expressed proteins) (Figure 2) were identified as expressed in RBC.  Figure 3(a) shows the protein-protein interaction network involving these 366 genes using the StringDB tool.

The functional analysis of the previous network showed that the most enriched KEGG pathways include the endocytosis pathway (hsa04144) (FDR = 9.83*e* − 10), the ubiquitin-mediated proteolysis (hsa04120) (FDR = 4.10*e* − 09), the focal adhesion pathway (hsa04510) (FDR = 6.74*e* − 07), the regulation of actin cytoskeleton pathway (hsa04810) with an FDR = 1.04*e* − 06, and the bacterial invasion of epithelial cells pathway (hsa05100) (FDR = 1.93*E* − 06) and the spliceosome (hsa03040) (FDR = 1.73*e* − 06). Reactome-enriched pathways include the immune system (FDR = 2.51*e* − 20), the membrane trafficking pathway (FDR = 6.36*e* − 20), the vesicle-mediated transport (FDR = 1.13*e* − 19), and the adaptive immune system (FDR = 7.95*e* − 15). Among the most enriched protein domains found with SMART and PFAM, we report “the ADP-ribosylation factor family” (FDR = 1.06*e* − 28), “the RAS, ROC, DAP kinase domain” (FDR = 1.01*e* − 25), “the Ras family” (FDR = 7.97*e* − 25) and the “Gtr1/RagA G protein conserved region” (FDR = 2.53*e* − 15).

On the other hand, among the 334 Plasmodium genes/proteins (involved in predicted interactions) (Figure 2), only 169 out of 2,430 Plasmodium RBC-expressed proteins were identified based on the integration of *P. falciparum* 3D7 expression data.  Figure 3(b) shows the protein-protein interaction network involving the 169 Plasmodium RBC-expressed proteins using the StringDB tool [42].

The functional analysis (using an FDR cutoff of 0.05) of this network showed that the most enriched KEGG pathways include malaria pathway (pfa05144) (FDR = 1.85*e* − 14, the metabolic pathway (pfa01100) (FDR = 0.0085), and the propanoate metabolism (pfa00640) (FDR = 0.0125). Among the most enriched Pfam domains, we report the PFEMP DBL domain (FDR = 1.35*E* − 22), the *N*-terminal segments of PfEMP1 (FDR = 2.11*E* − 21), the Duffy-binding domain (FDR = 7.18*E* − 21), and the acidic terminal segments, variant surface antigen of PfEMP1 (FDR = 1.09*E* − 20). Interestingly, Figure 3(b) showed that PFA0310c and FKBP35 play a key role and act like linkers between proteins involved in malaria pathways (represented in blue color in Figure 3(b)) and other proteins in the network. PFA0310c and FKBP35 encode for a P-type calcium transporting ATPase sarcoplasmic and endoplasmic reticulum Ca-ATPase, belonging to the cation transport ATPase (P-type) (TC 3.A.3) family and for a peptidylprolyl isomerase, FK506-binding protein- (FKBP-) type peptidylprolyl isomerase, respectively. These proteins may have essential roles in *P. falciparum* erythrocytic stages and may represent good parasite therapeutic targets.

### 3.3. The Human-*Plasmodium falciparum* 3D7 Interactome

**Figure 4:**
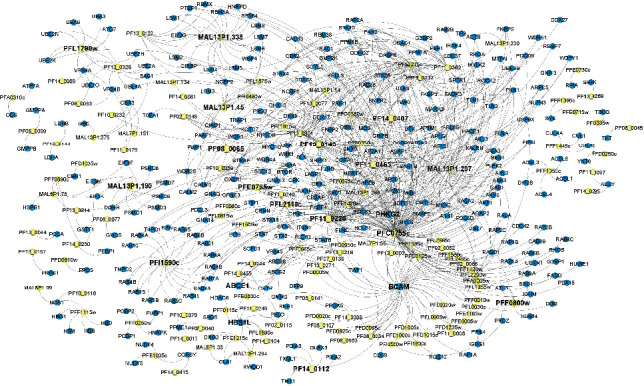
Host pathogen protein interaction network. Blue nodes denote human proteins. Yellow nodes refer to *Plasmodium falciparum* 3D7 proteins. The network was generated using Cytoscape 3.7.2.

The simultaneous integration of human and *Plasmodium falciparum* 3D7 expression data with the machine learning approach predictions allowed to identify 716 human-*Plasmodium falciparum* 3D7 interacting proteins in postinfection settings that involve 130 distinct parasite proteins and 302 unique human proteins (Figures 2 and 4, Supplementary Table 1).  Figure 4 displays a human-*Plasmodium falciparum* 3D7 interacting network following infection.

In order to assess the reliability of the human-Plasmodium interacting proteins reported in the current study, we have compared our results with previous experimental and computational initiatives that are aimed at deciphering human-*Plasmodium falciparum* interactions.

We found one interaction previously reported by [15]. This interaction involves PF14_0407 (PF3D7_1442900) encoding for a putative guanine nucleotide exchange factor with SAR1B (secretion associated Ras-related GTPase 1B).

Furthermore, four other interactions were previously reported by [20]. These later correspond to the interaction of PF14_0244 (PF3D7_1426500) (ABC transporter, putative) with ABCG2 (ATP-binding cassette subfamily G member 2 (Junior blood group)), MAL13P1.294 (PF3D7_1358900) (GTP binding protein, putative) with RWDD1 (RWD domain containing 1), PF13_0157 (PF3D7_1327800) (ribose-phosphate pyrophosphokinase, putative) with PRPS1 (phosphoribosyl pyrophosphate synthetase 1), and PFI0550w (PF3D7_0911300) (hypothetical protein) with human CD59 protein (CD59 molecule (CD59 blood group)). The same study reported an interaction between MAL13P1.190 (PF3D7_1338100) (proteasome regulatory component, putative) with PSMD4 that has not been detected in our study. However, we report novel interactions of this parasite protein with PSMD1, PSMD3, PSMD6, PSMD7, PSMD8, and others (Supplementary Table 1). Among interesting findings, we report no previously reported interactions related to Basal Cell Adhesion Molecule (BCAM) human protein that interacts with 43 Plasmodium proteins encoding for erythrocyte membrane protein 1 (PfEMP1), ABC transporter, (heavy metal transporter family), cytochrome C oxidase subunit, phosphatidylinositol 3-kinase, preprocathepsin c precursor, serine protease belonging to subtilisin family, and histone acetyltransferase GCN5 (Figure 4, Supplementary Table 1). Moreover, we report potential interactions between the human ATP-binding cassette transporter ABCB6 which has been shown to encode the Langereis (Lan) blood group antigen and Plasmodium proteins encoding for ABC transporters. We also reported potential binding of the human protein ICAM-4 to PFF0800w Plasmodium protein.

Moreover, among the 130 *Plasmodium falciparum* 3D7 genes involved in the predicted interactions, five were shown to be expressed exclusively in the ring stage, eight in the trophozoite stage, three in the schizont stage, and 19 in the merozoite stage. These genes are associated to 26, 75, 122, and 19 interactions, respectively (Supplementary Table 1). Out of these 130 *Plasmodium falciparum* 3D7 genes involved in the predicted interactions, we also identified 5 proteins (PF11_0245, PF13_0044, PF14_0230, PFE1035c, PFL0815w) expressed in both ring and trophozoite stages, two proteins (PF07_0139, PF08_0141) expressed in both trophozoites and merozoites, 1 protein (PF11_0097) expressed in both trophozoites and schizont, 1 protein (PF11_0362) expressed in both ring and schizont, and 32 expressed in the 4 stages (Supplementary Table 1). A functional analysis of these proteins involved in transition from a stage to another showed that these later are enriched in PFEMP DBL domain (PF03011) (FDR = 0.00066), Duffy-binding domain (PF05424) (FDR = 0.00066), acidic terminal segments, variant surface antigen of PfEMP1 (PF15445) (FDR = 0.00066), and N-terminal segments of PfEMP1 (PF15447) (FDR = 0.00066). These proteins may present a good therapeutic target to avoid parasite transition state and stop parasite life cycle progress.

## 4. Discussion

Host-pathogen protein-protein interactions underlie the critical process of infectious diseases by which pathogenic agents are able to invade host cells [51]. In the context of malaria disease, diverse efforts have been made during the past decades to identify human-Plasmodium proteins interaction including the use of computational methods to understand the mechanisms underlying the disease in order to develop novel therapeutic solutions [11, 12, 14, 17, 18]. While these investigations are reliable and represent highly valuable resources contributing to decipher the mechanisms underlying malaria disease, some may contain large false-positive predictions due to the exclusion of important criteria such as gene expression data in human and Plasmodium parasite and/or domain-domain interactions [12, 14, 15, 17, 18]. Indeed, gene expression data and domain-domain interactions constitute essential and key criteria that have to be considered. In order to comprehensively describe infections, the underlying gene expression changes in host and pathogen need to be clearly understood. Here, we are proposing an integrative computational approach based on the combination of multiple criteria including GO terms, similarity structures between proteins, homology data, domain-domain protein interactions, gene expression data in the host, and the pathogen and machine learning approaches. We used human and Plasmodium expression data previously identified by mass-spectroscopic proteome analysis of the human or plasmodium red blood cell that mainly relies on the physical separation of the two infection partners. While this technique is widely used, the dual RNAseq technique is another method that allows to profile gene expression in an infecting pathogen and its infected host simultaneously [52, 53] permitting a better investigation of host-pathogen proteins interaction when such data are available.

Subsequently, we report a set of human-*Plasmodium falciparum* 3D7 protein-protein interactions that have not been reported before including human proteins BCAM, ABCB6, and ICAM-4 with a considerable number of Plasmodium proteins known to be involved in the disease and expressed at different parasite stage life cycle. BCAM encodes for Lutheran blood group glycoprotein, a member of the immunoglobulin superfamily and a receptor for the extracellular matrix protein, laminin. Previous studies have identified BCAM as a receptor for Escherichia coli Cytotoxic Necrotizing Factor 1 (CNF1) and show that it is essential for cell intoxication [54]. A recent study assessed ABCB6 as a host factor for *Plasmodium falciparum* malaria parasites during erythrocyte invasion and that ABCB6 may mediate *P. falciparum* invasion through species protein-protein molecular interactions [55]. Moreover, it was previously suggested that ICAM-4 binds to *P. falciparum* merozoites, and the addition of recombinant ICAM-4 to parasite cultures blocks invasion of erythrocytes by newly released merozoites [56]. BCAM is an extracellular matrix protein belonging to the immunoglobulin superfamily. It interacts with laminin via its five immunoglobulin-like domains. On the other hand, PfEMP1 has been demonstrated to use ICAM-1, another surface protein belonging to the immunoglobulin superfamily, to interact with the host red cell [57]. Given the homology between ICAM-1 and BCAM, it is therefore likely that they would contribute to the same biological process in the physiopathology of the *P. falciparum* infection. The existing overlap with previous studies consolidates the reliability and credibility of the present approach that could be applied to investigate other host-pathogen protein-protein interactions. We reported a set of proteins involved in transition from parasite stage to another including PFE1035c and PF13_0044 that are enriched in pyrimidine metabolism KEGG pathway and in PFEMP DBL domain (PF03011) (FDR = 0.00066), Duffy-binding domain (PF05424) (FDR = 0.00066), acidic terminal segments, variant surface antigen of PfEMP1 (PF15445) (FDR = 0.00066), and N-terminal segments of PfEMP1 (PF15447) (FDR = 0.00066). These proteins may present a good therapeutic target to avoid parasite transition state and stop parasite life cycle progress. A previous study reported the important role of the purine and pyrimidine pathways for *P. falciparum* cell growth and division. Indeed, the rapid rate of nucleic acid synthesis during the intraerythrocytic growth phase makes purine and pyrimidine metabolic pathways promising targets for novel drug development [58]. Furthermore, we reported another protein PF11_0240 encoding for dynein heavy chain that may present an interesting therapeutic target. Another study investigated the role of dynein heavy chain, suggesting that it may play a role in the flagellar motility of the male gametes [59]. Moreover, we reported some *Plasmodium falciparum* hub proteins (Figure 4) having the potential to interact with several human proteins including PFI0480w encoding for a helicase with Zn-finger motif, PF11_0240 that encodes for a dynein heavy chain, and PFE0765w encoding for a phosphatidylinositol 3-kinase. Additional studies have shown that helicases are omnipresent enzymes playing a prominent role in nucleic acid metabolism and can be used as potential targets for the development of novel therapeutics [60]. In addition, it was previously shown that the inhibition of phosphatidylinositol 3-kinase prevented the parasite transport to the food vacuole, the site of hemoglobin catabolism, and caused the inhibition of parasite growth [61].

## 5. Conclusions

Computational methods may play important roles in paving the way for experimental host-pathogen interactions verification by highlighting key potential interactions and limiting the experimental scope leading to expense reduction and rapid knowledge generation. Here, we investigated human-*Plasmodium* protein-protein interactions using an integrative computational approach. We report a set of biologically relevant host-pathogen interactions that will enrich existing resources and may contribute to a better understanding of the etiology of the disease. The present approach is not restricted to a particular host or pathogen but can be applied for predicting other host-pathogen interactions unless gene expression data is available. The detailed interaction map between proteins from the pathogen and the human host would help to identify key hubs in the infection physiopathology process.

## Figures and Tables

**Table 1:** Classifiers evaluated performance and metrics.

Model	Accuracy	Precision	Recall	F1-score	AUC score	FPR
VC	94	94	94	94	98	7
SVM	93	93	93	93	98	10
LR	91	91	91	90	93	16
KNN	88	88	88	88	88	14
Adaboost	85	85	85	85	92	15
RF	82	85	82	81	92	21
GNB	74	75	74	75	81	20
DTree	74	74	74	74	72	22

VC: voting classifier; SVM: support vector machine; LR: logistic regression; KNN: *K*-nearest neighbor; RF: random forest; GNB: Gaussian Naive Bayes; DTree: decision tree.

## Data Availability

All data and results are provided within the manuscript or in the supplementary table provided online with the manuscript.

## Supplementary Materials

Supplementary 1 ROC curves (receiver operating characteristic curves) showing the performance of investigated classifiers.

Supplementary 2 Performance evaluation of investigated classifiers.

Supplementary 3 Table 1: human-*Plasmodium falciparum* 3D7 protein-protein interactions predicted by the present integrative computational approach.
