# Supplementary figures and images for: An Integrative Computational Approach for the Prediction of Human-Plasmodium Protein-Protein Interactions
Source: Biomed Res Int. 2020 Dec 19;2020:2082540. doi: 10.1155/2020/2082540 (PMC7771252; doi:10.1155/2020/2082540)

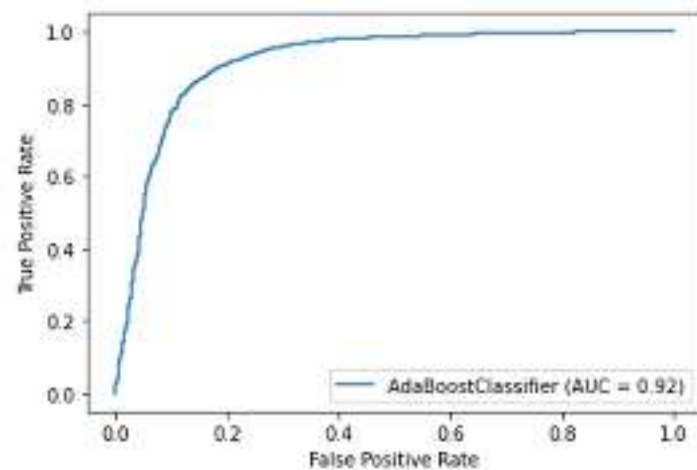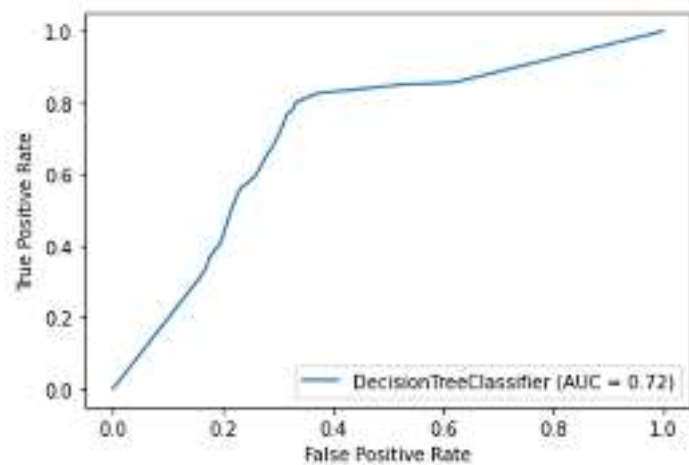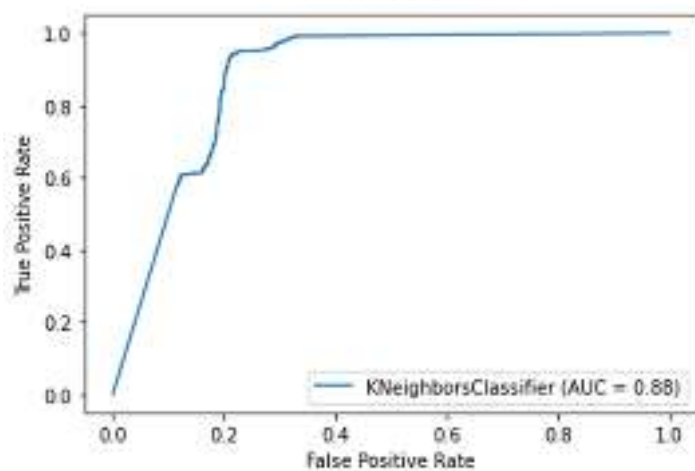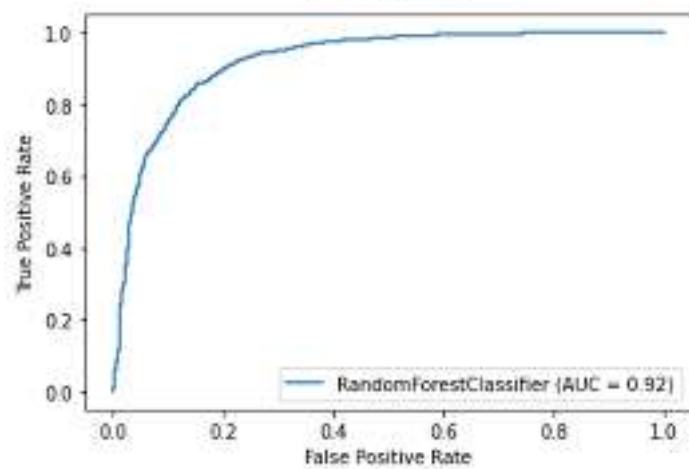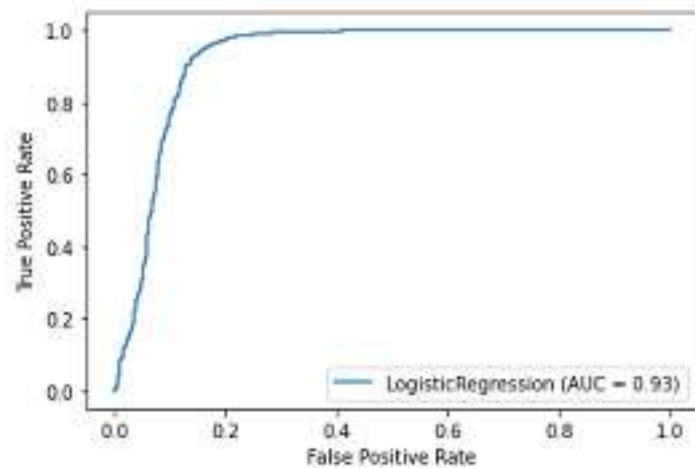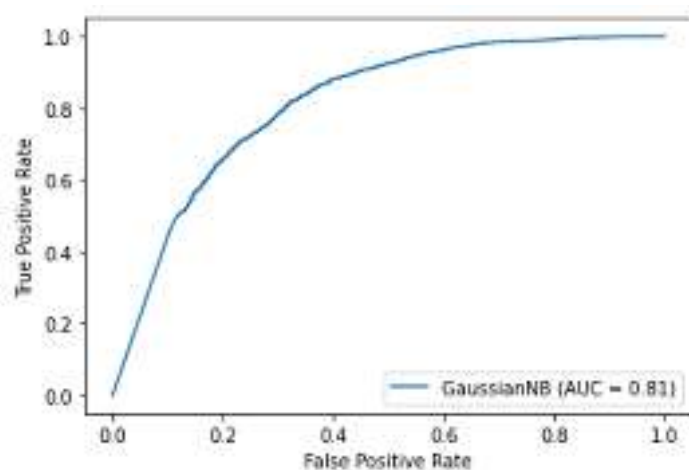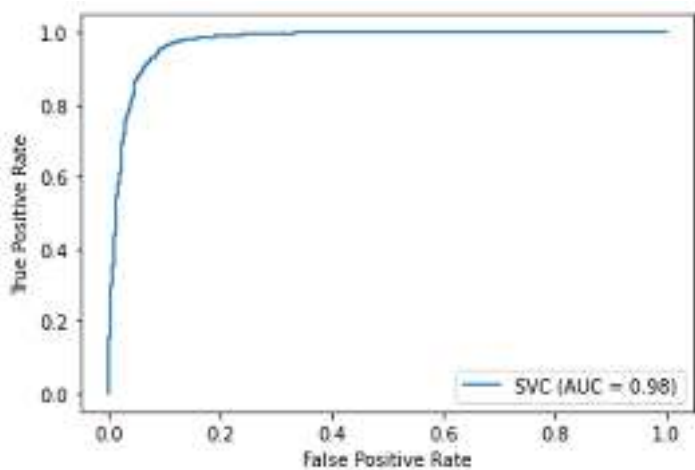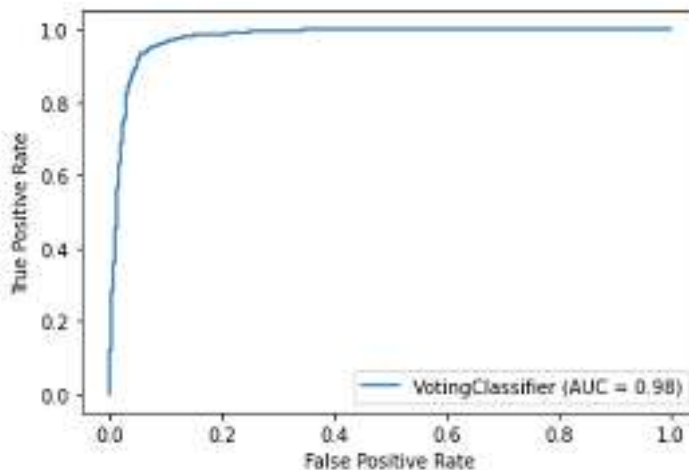

Supplement: Supplementary 1 — ROC curves (receiver operating characteristic curves) showing the performance of investigated classifiers. [file 2082540.f1.pdf]

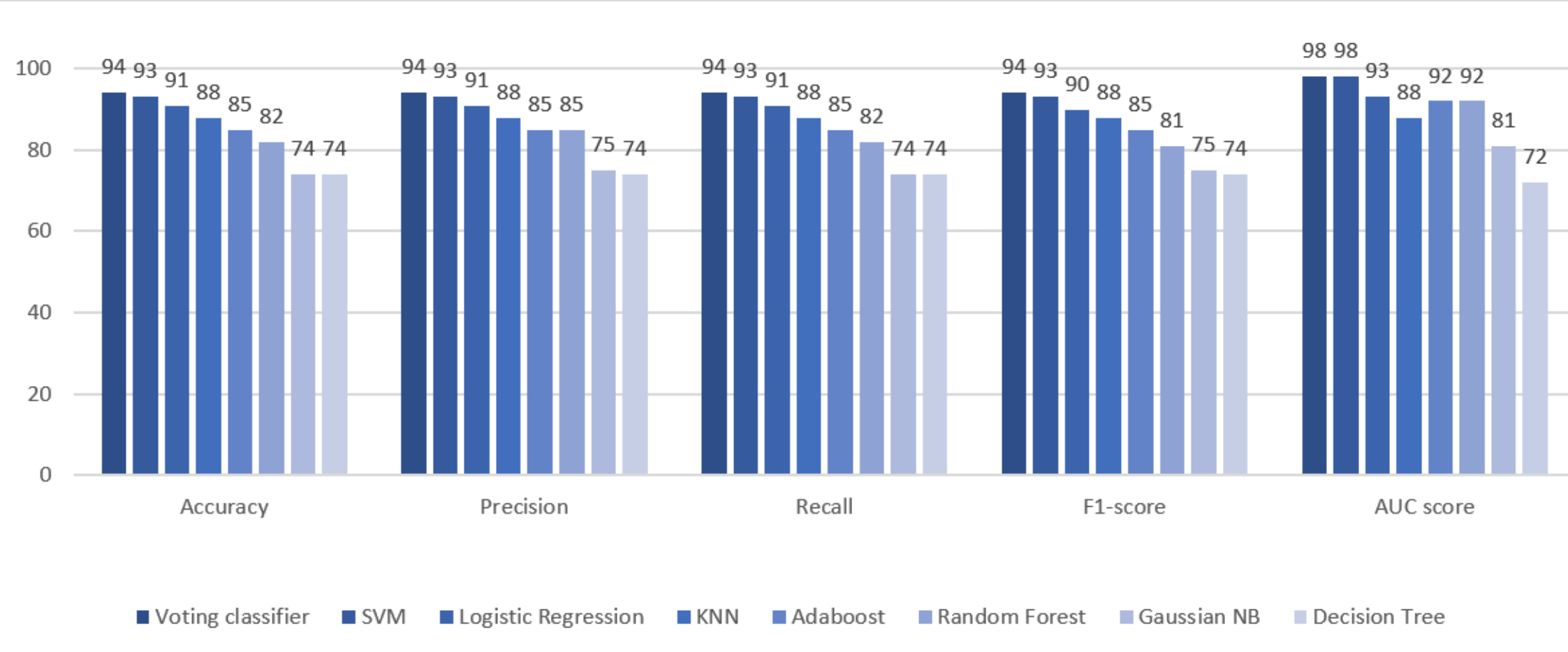

Supplement: Supplementary 2 — Performance evaluation of investigated classifiers. [file 2082540.f2.pdf]
