# Supplementary material for: An Integrative Computational Approach for the Prediction of Human-Plasmodium Protein-Protein Interactions
Source: Biomed Res Int. 2020 Dec 19;2020:2082540. doi: 10.1155/2020/2082540 (PMC7771252; doi:10.1155/2020/2082540)
Supplement: Supplementary 3 — Table 1: human-Plasmodium falciparum 3D7 protein-protein interactions predicted by the present integrative computational approach. [file 2082540.f3.pdf]

Supplementary Table 1 : Human-Plasmodium falciparum 3D7 protein Protein interactions predicted by the present integrative computational Approach.

Symbol	Description	PF ID	Aliases	Description	Expression by stage	GO term
ABCB6	ATP binding cassette subfamily B member 6 (Langereis blood group)	PF3D7_0302600	PFC0125w	ABC transporter, putative	Gametocytes	
ABCB6	ATP binding cassette subfamily B member 6 (Langereis blood group)	PF3D7_1339900	PF13_0218			
ABCB6	ATP binding cassette subfamily B member 6 (Langereis blood group)	PF3D7_1352100	PF13_0271	ABC transporter, (heavy metal transporter family) [Plasmodium falciparum 3D7]	Global	GO:0006810
ABCE1	ATP binding cassette subfamily E member 1	PF3D7_0212300	PF02_0115			
ABCE1	ATP binding cassette subfamily E member 1	PF3D7_0302600	PFC0125w	ABC transporter, putative	Gametocytes	
ABCE1	ATP binding cassette subfamily E member 1	PF3D7_1339900	PF13_0218			
ABCE1	ATP binding cassette subfamily E member 1	PF3D7_1352100	PF13_0271	ABC transporter, (heavy metal transporter family) [Plasmodium falciparum 3D7]	Global	GO:0006810
ABCE1	ATP binding cassette subfamily E member 1	PF3D7_1426500	PF14_0244	ABC transporter, putative	Gametocytes	
ABCE1	ATP binding cassette subfamily E member 1	PF3D7_1447900	PF14_0455			

ABCG2	ATP binding cassette subfamily G member 2 (Junior blood group)	PF3D7_0302600	PFC0125w	ABC transporter, putative	Gametocytes	
ABCG2	ATP binding cassette subfamily G member 2 (Junior blood group)	PF3D7_1339900	PF13_0218			
ABCG2	ATP binding cassette subfamily G member 2 (Junior blood group)	PF3D7_1352100	PF13_0271	ABC transporter, (heavy metal transporter family) [Plasmodium falciparum 3D7]	Global	GO:0006810
ABCG2	ATP binding cassette subfamily G member 2 (Junior blood group)	PF3D7_1426500	PF14_0244	ABC transporter, putative	Gametocytes	
ABCG2	ATP binding cassette subfamily G member 2 (Junior blood group)	PF3D7_1447900	PF14_0455			
ACAP2	ArfGAP with coiled-coil, ankyrin repeat and PH domains 2	PF3D7_0309000	PFC0380w	dual-specificity protein phosphatase, putative	Troph/ Gametocytes	
ACAP2	ArfGAP with coiled-coil, ankyrin repeat and PH domains 2	PF3D7_0317200	PFC0755c	protein kinase, putative [Plasmodium falciparum 3D7]	Schizont	GO:0006468
ACAP2	ArfGAP with coiled-coil, ankyrin repeat and PH domains 2	PF3D7_0528500	PFE1420w			
ACAP2	ArfGAP with coiled-coil, ankyrin repeat and PH domains 2	PF3D7_1121300	PF11_0220	hypothetical protein	Troph/ Gametocytes	

ACAP2	ArfGAP with coiled-coil, ankyrin repeat and PH domains 2	PF3D7_1247800	PFL2290w	preprocathepsin c precursor, putative	Troph/ Gametocytes	
ACAP2	ArfGAP with coiled-coil, ankyrin repeat and PH domains 2	PF3D7_1329300	PF13_0149	chromatin assembly factor 1 subunit, putative [Plasmodium falciparum 3D7]	Global	GO:0006333
ACAP2	ArfGAP with coiled-coil, ankyrin repeat and PH domains 2	PF3D7_1359800	MAL13P1.297			
ACAP2	ArfGAP with coiled-coil, ankyrin repeat and PH domains 2	PF3D7_1442900	PF14_0407	guanine nucleotide exchange factor, putative [Plasmodium falciparum 3D7]	Ring	GO:0032012
ACLY	ATP citrate lyase	PF3D7_1108500	PF11_0097	succinyl-CoA synthetase alpha subunit, putative [Plasmodium falciparum 3D7]	Troph/ Schizont	GO:0006104
ACLY	ATP citrate lyase	PF3D7_1431600	PF14_0295	ATP-specific succinyl-CoA synthetase beta subunit, putative [Plasmodium falciparum 3D7]	Global	GO:0006104
ACSL1	acyl-CoA synthetase long chain family member 1	PF3D7_0627800	PFF1350c			
ACSL3	acyl-CoA synthetase long chain family member 3	PF3D7_0627800	PFF1350c			

ACSL4	acyl-CoA synthetase long chain family member 4	PF3D7_0627800	PFF1350c			
ACSL6	acyl-CoA synthetase long chain family member 6	PF3D7_0627800	PFF1350c			
ACTB	actin beta	PF3D7_0317200	PFC0755c	protein kinase, putative [Plasmodium falciparum 3D7]	Schizont	GO:0006468
ACTB	actin beta	PF3D7_0528500	PFE1420w			
ACTB	actin beta	PF3D7_0613900	PFF0675c	myosin E [Plasmodium falciparum 3D7]	Schizont	GO:0003774
ACTB	actin beta	PF3D7_1346000	MAL13P1.230	hypothetical protein	Gametocytes	
AGFG1	ArfGAP with FG repeats 1	PF3D7_1359800	MAL13P1.297			
ANK1	ankyrin 1	PF3D7_0317200	PFC0755c	protein kinase, putative [Plasmodium falciparum 3D7]	Schizont	GO:0006468
ANK1	ankyrin 1	PF3D7_1359800	MAL13P1.297			
ANK3	ankyrin 3	PF3D7_0309000	PFC0380w	dual-specificity protein phosphatase, putative	Troph/ Gametocytes	
ANK3	ankyrin 3	PF3D7_0317200	PFC0755c	protein kinase, putative [Plasmodium falciparum 3D7]	Schizont	GO:0006468
ANK3	ankyrin 3	PF3D7_0528500	PFE1420w			
ANK3	ankyrin 3	PF3D7_1247800	PFL2290w	preprocathepsin c precursor, putative	Troph/ Gametocytes	
ANK3	ankyrin 3	PF3D7_1358000	MAL13P1.285	patatin-like phospholipase, putative [Plasmodium falciparum 3D7]	Global	GO:0006629

ANK3	ankyrin 3	PF3D7_1359800	MAL13P1.297			
AP1B1	adaptor related protein complex 1 subunit beta 1	PF3D7_1145100	PF11_0463	coat protein, gamma subunit, putative [Plasmodium falciparum 3D7]	Global	GO:0006886
AP1B1	adaptor related protein complex 1 subunit beta 1	PF3D7_1359800	MAL13P1.297			
AP1G1	adaptor related protein complex 1 subunit gamma 1	PF3D7_1145100	PF11_0463	coat protein, gamma subunit, putative [Plasmodium falciparum 3D7]	Global	GO:0006886
AP1G1	adaptor related protein complex 1 subunit gamma 1	PF3D7_1359800	MAL13P1.297			
AP1M1	adaptor related protein complex 1 subunit mu 1	PF3D7_1359800	MAL13P1.297			
AP2A1	adaptor related protein complex 2 subunit alpha 1	PF3D7_1145100	PF11_0463	coat protein, gamma subunit, putative [Plasmodium falciparum 3D7]	Global	GO:0006886
AP2A2	adaptor related protein complex 2 subunit alpha 2	PF3D7_1145100	PF11_0463	coat protein, gamma subunit, putative [Plasmodium falciparum 3D7]	Global	GO:0006886
AP2A2	adaptor related protein complex 2 subunit alpha 2	PF3D7_1359800	MAL13P1.297			

AP2B1	adaptor related protein complex 2 subunit beta 1	PF3D7_1145 100	PF11_0463	coat protein, gamma subunit, putative [Plasmodium falciparum 3D7]	Global	GO:0006886
AP2B1	adaptor related protein complex 2 subunit beta 1	PF3D7_1359 800	MAL13P1.297			
AP2M1	adaptor related protein complex 2 subunit mu 1	PF3D7_1359 800	MAL13P1.297			
ARAF	A-Raf proto-oncogene, serine/threon ine kinase	PF3D7_0317 200	PFC0755c	protein kinase, putative [Plasmodium falciparum 3D7]	Schizont	GO:0006468
ARAF	A-Raf proto-oncogene, serine/threon ine kinase	PF3D7_1121 300	PF11_0220	hypothetical protein	Troph/ Gametocytes	
ARAF	A-Raf proto-oncogene, serine/threon ine kinase	PF3D7_1243 900	PFL2110c	conserved protein [Plasmodium falciparum 3D7]		
ARAF	A-Raf proto-oncogene, serine/threon ine kinase	PF3D7_1247 400	PFL2275c	FK506-binding protein (FKBP)-type peptidyl-propyl isomerase [Plasmodium falciparum 3D7]	Troph	GO:0006457
ARF1	ADP ribosylation factor 1	PF3D7_1359 800	MAL13P1.297			
ARF6	ADP ribosylation factor 6	PF3D7_1359 800	MAL13P1.297			
ARL2	ADP ribosylation factor like GTPase 2	PF3D7_1359 800	MAL13P1.297			

ARL2	ADP ribosylation factor like GTPase 2	PF3D7_1442900	PF14_0407	guanine nucleotide exchange factor, putative [Plasmodium falciparum 3D7]	Ring	GO:0032012
ARL3	ADP ribosylation factor like GTPase 3	PF3D7_1359800	MAL13P1.297			
ARL8B	ADP ribosylation factor like GTPase 8B	PF3D7_1359800	MAL13P1.297			
ARPC5	actin related protein 2/3 complex subunit 5	PF3D7_1329300	PF13_0149	chromatin assembly factor 1 subunit, putative [Plasmodium falciparum 3D7]	Global	GO:0006333
ASAP1	ArfGAP with SH3 domain, ankyrin repeat and PH domain 1	PF3D7_0309000	PFC0380w	dual-specificity protein phosphatase, putative	Troph/ Gametocytes	
ASAP1	ArfGAP with SH3 domain, ankyrin repeat and PH domain 1	PF3D7_0317200	PFC0755c	protein kinase, putative [Plasmodium falciparum 3D7]	Schizont	GO:0006468
ASAP1	ArfGAP with SH3 domain, ankyrin repeat and PH domain 1	PF3D7_0528500	PFE1420w			
ASAP1	ArfGAP with SH3 domain, ankyrin repeat and PH domain 1	PF3D7_1121300	PF11_0220	hypothetical protein	Troph/ Gametocytes	
ASAP1	ArfGAP with SH3 domain, ankyrin repeat and PH domain 1	PF3D7_1247800	PFL2290w	preprocathepsin c precursor, putative	Troph/ Gametocytes	
ASAP1	ArfGAP with SH3 domain, ankyrin repeat and PH domain 1	PF3D7_1329300	PF13_0149	chromatin assembly factor 1 subunit, putative [Plasmodium falciparum 3D7]	Global	GO:0006333

ASAP1	ArfGAP with SH3 domain, ankyrin repeat and PH domain 1	PF3D7_1359800	MAL13P1.297			
ASAP1	ArfGAP with SH3 domain, ankyrin repeat and PH domain 1	PF3D7_1442900	PF14_0407	guanine nucleotide exchange factor, putative [Plasmodium falciparum 3D7]	Ring	GO:0032012
ATG7	autophagy related 7	PF3D7_1237000	PFL1790w	ubiquitin-activating enzyme, putative [Plasmodium falciparum 3D7]	Global	GO:0006464
ATL3	atlastin GTPase 3	PF3D7_0317200	PFC0755c	protein kinase, putative [Plasmodium falciparum 3D7]	Schizont	GO:0006468
ATL3	atlastin GTPase 3	PF3D7_0616500	PFF0800w			
ATP7A	ATPase copper transporting alpha	PF3D7_0106300	PFA0310c	calcium-transporting ATPase, putative [Plasmodium falciparum 3D7]	Global	GO:0006754
BAG1	BAG cochaperone 1	PF3D7_0317200	PFC0755c	protein kinase, putative [Plasmodium falciparum 3D7]	Schizont	GO:0006468
BAG1	BAG cochaperone 1	PF3D7_1355700	MAL13P1.275	protein phosphatase, putative [Plasmodium falciparum 3D7]	Global	None
BAG1	BAG cochaperone 1	PF3D7_1361900	PF13_0328	proliferating cell nuclear antigen [Plasmodium falciparum 3D7]	Troph	GO:0006275
BBS1	Bardet-Biedl syndrome 1	PF3D7_1359800	MAL13P1.297			

BCAM	basal cell adhesion molecule (Lutheran blood group)	PF3D7_0100100	PFA0005w	erythrocyte membrane protein 1 (PfEMP1)	Merozoites	
BCAM	basal cell adhesion molecule (Lutheran blood group)	PF3D7_0207000	PF02_0066			
BCAM	basal cell adhesion molecule (Lutheran blood group)	PF3D7_0208600	PF02_0082			
BCAM	basal cell adhesion molecule (Lutheran blood group)	PF3D7_0302600	PFC0125w	ABC transporter, putative	Gametocytes	
BCAM	basal cell adhesion molecule (Lutheran blood group)	PF3D7_0317200	PFC0755c	protein kinase, putative [Plasmodium falciparum 3D7]	Schizont	GO:0006468
BCAM	basal cell adhesion molecule (Lutheran blood group)	PF3D7_0321500	PFC0950c	peptidase, putative [Plasmodium falciparum 3D7]	Troph	GO:0006508
BCAM	basal cell adhesion molecule (Lutheran blood group)	PF3D7_0400100	PFD0005w	erythrocyte membrane protein 1 (PfEMP1)	Troph	
BCAM	basal cell adhesion molecule (Lutheran blood group)	PF3D7_0400400	PFD0020c	erythrocyte membrane protein 1 (PfEMP1)	Merozoites	
BCAM	basal cell adhesion molecule (Lutheran blood group)	PF3D7_0412700	PFD0625c			
BCAM	basal cell adhesion molecule (Lutheran blood group)	PF3D7_0420700	PFD0995c	erythrocyte membrane protein 1 (PfEMP1)	Merozoites	
BCAM	basal cell adhesion molecule (Lutheran blood group)	PF3D7_0420900	PFD1000c	erythrocyte membrane protein 1 (PfEMP1)	Merozoites	

BCAM	basal cell adhesion molecule (Lutheran blood group)	PF3D7_0421100	PFD1005c	erythrocyte membrane protein 1 (PfEMP1)	Merozoites	
BCAM	basal cell adhesion molecule (Lutheran blood group)	PF3D7_0421300	PFD1015c	erythrocyte membrane protein 1 (PfEMP1)	Merozoites/ Gametocytes	
BCAM	basal cell adhesion molecule (Lutheran blood group)	PF3D7_0425800	PFD1235w	erythrocyte membrane protein 1 (PfEMP1)	Merozoites/ Gametocytes	
BCAM	basal cell adhesion molecule (Lutheran blood group)	PF3D7_0500100	PFE0005w	erythrocyte membrane protein 1 (PfEMP1)	Merozoites	
BCAM	basal cell adhesion molecule (Lutheran blood group)	PF3D7_0523800	PFE1185w	transporter, putative	Merozoites	
BCAM	basal cell adhesion molecule (Lutheran blood group)	PF3D7_0600200	PFF0010w			
BCAM	basal cell adhesion molecule (Lutheran blood group)	PF3D7_0616500	PFF0800w			
BCAM	basal cell adhesion molecule (Lutheran blood group)	PF3D7_0629300	PFF1420w	phosphatidylcholine-sterol acyltransferase precursor, putative [Plasmodium falciparum 3D7]	Unknown	GO:0006629
BCAM	basal cell adhesion molecule (Lutheran blood group)	PF3D7_0632500	PFF1580c			
BCAM	basal cell adhesion molecule (Lutheran blood group)	PF3D7_0632800	PFF1595c			
BCAM	basal cell adhesion molecule (Lutheran blood group)	PF3D7_0712800	MAL7P1.55	erythrocyte membrane protein 1 (PfEMP1)	Gametocytes	

BCAM	basal cell adhesion molecule (Lutheran blood group)	PF3D7_0733 000	PF07_0139	erythrocyte membrane protein 1 (PfEMP1)	Merozoites/ Troph	
BCAM	basal cell adhesion molecule (Lutheran blood group)	PF3D7_0800 200	PF08_0141	erythrocyte membrane protein 1 (PfEMP1)	Merozoites/ Troph	
BCAM	basal cell adhesion molecule (Lutheran blood group)	PF3D7_0808 600	PF08_0107	erythrocyte membrane protein 1 (PfEMP1)	Merozoites	
BCAM	basal cell adhesion molecule (Lutheran blood group)	PF3D7_0809 100	PF08_0103	erythrocyte membrane protein 1 (PfEMP1)	Merozoites	
BCAM	basal cell adhesion molecule (Lutheran blood group)	PF3D7_0823 300	PF08_0034	histone acetyltransfe rase GCN5, putative [Plasmodium falciparum 3D7]	Global	GO:0006355
BCAM	basal cell adhesion molecule (Lutheran blood group)	PF3D7_0911 300	PFI0550w	hypothetical protein	Sporozoites/ Merozoites	
BCAM	basal cell adhesion molecule (Lutheran blood group)	PF3D7_0937 800	PFI1830c	pfEMP1	Troph/ Gametocytes	
BCAM	basal cell adhesion molecule (Lutheran blood group)	PF3D7_1100 200	PF11_0008	erythrocyte membrane protein 1 (PfEMP1)	Merozoites	
BCAM	basal cell adhesion molecule (Lutheran blood group)	PF3D7_1200 100	PFL0005w	erythrocyte membrane protein 1 (PfEMP1)	Merozoites	
BCAM	basal cell adhesion molecule (Lutheran blood group)	PF3D7_1200 400	PFL0020w	erythrocyte membrane protein 1 (PfEMP1)	Merozoites	
BCAM	basal cell adhesion molecule (Lutheran blood group)	PF3D7_1200 600	PFL0030c	erythrocyte membrane protein 1 (PfEMP1)	Merozoites	

BCAM	basal cell adhesion molecule (Lutheran blood group)	PF3D7_1240400	PFL1955w	erythrocyte membrane protein 1 (PfEMP1)	Gametocytes	
BCAM	basal cell adhesion molecule (Lutheran blood group)	PF3D7_1247800	PFL2290w	preprocathepsin c precursor, putative	Troph/ Gametocytes	
BCAM	basal cell adhesion molecule (Lutheran blood group)	PF3D7_1251700	PFL2485c			
BCAM	basal cell adhesion molecule (Lutheran blood group)	PF3D7_1255200	PFL2665c	erythrocyte membrane protein 1 (PfEMP1)	Troph/ Gametocytes	
BCAM	basal cell adhesion molecule (Lutheran blood group)	PF3D7_1300300	PF13_0003	erythrocyte membrane protein 1 (PfEMP1)	Merozoites	
BCAM	basal cell adhesion molecule (Lutheran blood group)	PF3D7_1329300	PF13_0149	chromatin assembly factor 1 subunit, putative [Plasmodium falciparum 3D7]	Global	GO:0006333
BCAM	basal cell adhesion molecule (Lutheran blood group)	PF3D7_1339900	PF13_0218			
BCAM	basal cell adhesion molecule (Lutheran blood group)	PF3D7_1352100	PF13_0271	ABC transporter, (heavy metal transporter family) [Plasmodium falciparum 3D7]	Global	GO:0006810
BCAM	basal cell adhesion molecule (Lutheran blood group)	PF3D7_1411400	PF14_0112			
BCAM	basal cell adhesion molecule (Lutheran blood group)	PF3D7_1430900	PF14_0288	cytochrome C oxidase subunit II precursor, putative	Gametocytes	

BMP2K	BMP2 inducible kinase	PF3D7_0309 000	PFC0380w	dual-specificity protein phosphatase, putative	Troph/ Gametocytes	
BMP2K	BMP2 inducible kinase	PF3D7_0317 200	PFC0755c	protein kinase, putative [Plasmodium falciparum 3D7]	Schizont	GO:0006468
BMP2K	BMP2 inducible kinase	PF3D7_1030 800	PF10_0301	calmodulin, putative [Plasmodium falciparum 3D7]	Global	GO:0005509
BMP2K	BMP2 inducible kinase	PF3D7_1121 300	PF11_0220	hypothetical protein	Troph/ Gametocytes	
BMP2K	BMP2 inducible kinase	PF3D7_1135 100	PF11_0362	protein phosphatase, putative [Plasmodium falciparum 3D7]	Ring/Schizont	None
BMP2K	BMP2 inducible kinase	PF3D7_1247 400	PFL2275c	FK506-binding protein (FKBP)-type peptidyl-propyl isomerase [Plasmodium falciparum 3D7]	Troph	GO:0006457
BMP2K	BMP2 inducible kinase	PF3D7_1342 400	PF13_0232	casein kinase II beta chain [Plasmodium falciparum 3D7]	Troph	GO:0004674
BMPR2	bone morphogenetic protein receptor type 2	PF3D7_0309 000	PFC0380w	dual-specificity protein phosphatase, putative	Troph/ Gametocytes	
BMPR2	bone morphogenetic protein receptor type 2	PF3D7_0317 200	PFC0755c	protein kinase, putative [Plasmodium falciparum 3D7]	Schizont	GO:0006468
BMPR2	bone morphogenetic protein receptor type 2	PF3D7_1121 300	PF11_0220	hypothetical protein	Troph/ Gametocytes	

BMPR2	bone morphogenetic protein receptor type 2	PF3D7_1135100	PF11_0362	protein phosphatase, putative [Plasmodium falciparum 3D7]	Ring/Schizont	None
BMPR2	bone morphogenetic protein receptor type 2	PF3D7_1247400	PFL2275c	FK506-binding protein (FKBP)-type peptidyl-propyl isomerase [Plasmodium falciparum 3D7]	Troph	GO:0006457
BMPR2	bone morphogenetic protein receptor type 2	PF3D7_1342400	PF13_0232	casein kinase II beta chain [Plasmodium falciparum 3D7]	Troph	GO:0004674
BTK	Bruton tyrosine kinase	PF3D7_0317200	PFC0755c	protein kinase, putative [Plasmodium falciparum 3D7]	Schizont	GO:0006468
BTK	Bruton tyrosine kinase	PF3D7_0515300	PFE0765w	phosphatidylinositol 3-kinase, putative [Plasmodium falciparum 3D7]	Global	GO:0006897
BTK	Bruton tyrosine kinase	PF3D7_1121300	PF11_0220	hypothetical protein	Troph/ Gametocytes	
BTK	Bruton tyrosine kinase	PF3D7_1243900	PFL2110c	conserved protein [Plasmodium falciparum 3D7]		
BTK	Bruton tyrosine kinase	PF3D7_1247400	PFL2275c	FK506-binding protein (FKBP)-type peptidyl-propyl isomerase [Plasmodium falciparum 3D7]	Troph	GO:0006457

BTK	Bruton tyrosine kinase	PF3D7_1329300	PF13_0149	chromatin assembly factor 1 subunit, putative [Plasmodium falciparum 3D7]	Global	GO:0006333
BTK	Bruton tyrosine kinase	PF3D7_1359800	MAL13P1.297			
BTK	Bruton tyrosine kinase	PF3D7_1442900	PF14_0407	guanine nucleotide exchange factor, putative [Plasmodium falciparum 3D7]	Ring	GO:0032012
CAB39	calcium binding protein 39	PF3D7_0317200	PFC0755c	protein kinase, putative [Plasmodium falciparum 3D7]	Schizont	GO:0006468
CALM1	calmodulin 1	PF3D7_0317200	PFC0755c	protein kinase, putative [Plasmodium falciparum 3D7]	Schizont	GO:0006468
CALM1	calmodulin 1	PF3D7_1030800	PF10_0301	calmodulin, putative [Plasmodium falciparum 3D7]	Global	GO:0005509
CALM1	calmodulin 1	PF3D7_1238700	PFL1875w	conserved Plasmodium protein [Plasmodium falciparum 3D7]	Unknown	GO:0006813
CALM1	calmodulin 1	PF3D7_1247400	PFL2275c	FK506-binding protein (FKBP)-type peptidyl-propyl isomerase [Plasmodium falciparum 3D7]	Troph	GO:0006457
CALM2	calmodulin 2	PF3D7_0317200	PFC0755c	protein kinase, putative [Plasmodium falciparum 3D7]	Schizont	GO:0006468

CALM2	calmodulin 2	PF3D7_1030800	PF10_0301	calmodulin, putative [Plasmodium falciparum 3D7]	Global	GO:0005509
CALM2	calmodulin 2	PF3D7_1238700	PFL1875w	conserved Plasmodium protein [Plasmodium falciparum 3D7]	Unknown	GO:0006813
CALM2	calmodulin 2	PF3D7_1247400	PFL2275c	FK506-binding protein (FKBP)-type peptidyl-propyl isomerase [Plasmodium falciparum 3D7]	Troph	GO:0006457
CALM3	calmodulin 3	PF3D7_0317200	PFC0755c	protein kinase, putative [Plasmodium falciparum 3D7]	Schizont	GO:0006468
CALM3	calmodulin 3	PF3D7_1030800	PF10_0301	calmodulin, putative [Plasmodium falciparum 3D7]	Global	GO:0005509
CALM3	calmodulin 3	PF3D7_1238700	PFL1875w	conserved Plasmodium protein [Plasmodium falciparum 3D7]	Unknown	GO:0006813
CALM3	calmodulin 3	PF3D7_1247400	PFL2275c	FK506-binding protein (FKBP)-type peptidyl-propyl isomerase [Plasmodium falciparum 3D7]	Troph	GO:0006457
CAPZB	capping actin protein of muscle Z-line subunit beta	PF3D7_0528500	PFE1420w			

CASP8	caspase 8	PF3D7_0317200	PFC0755c	protein kinase, putative [Plasmodium falciparum 3D7]	Schizont	GO:0006468
CBR1	carbonyl reductase 1	PF3D7_0422000	PFD1035w	steroid dehydrogenase, putative [Plasmodium falciparum 3D7]	Global	GO:0006633
CBX3	chromobox 3	PF3D7_0727300	MAL7P1.151			
CBX3	chromobox 3	PF3D7_1023900	PF10_0232	Chromodomain-helicase-DNA-binding protein 1 homolog, putative [Plasmodium falciparum 3D7]	Global	GO:0006333
CCS	copper chaperone for superoxide dismutase	PF3D7_0106300	PFA0310c	calcium-transporting ATPase, putative [Plasmodium falciparum 3D7]	Global	GO:0006754
CD2AP	CD2 associated protein	PF3D7_1121300	PF11_0220	hypothetical protein	Troph/ Gametocytes	
CD59	CD59 molecule (CD59 blood group)	PF3D7_0911300	PFI0550w	hypothetical protein	Sporozoites/ Merozoites	
CDC37	cell division cycle 37	PF3D7_0317200	PFC0755c	protein kinase, putative [Plasmodium falciparum 3D7]	Schizont	GO:0006468
CDC42	cell division cycle 42	PF3D7_0317200	PFC0755c	protein kinase, putative [Plasmodium falciparum 3D7]	Schizont	GO:0006468
CDK2	cyclin dependent kinase 2	PF3D7_0309000	PFC0380w	dual-specificity protein phosphatase, putative	Troph/ Gametocytes	

CDK2	cyclin dependent kinase 2	PF3D7_0317200	PFC0755c	protein kinase, putative [Plasmodium falciparum 3D7]	Schizont	GO:0006468
CDK2	cyclin dependent kinase 2	PF3D7_1030800	PF10_0301	calmodulin, putative [Plasmodium falciparum 3D7]	Global	GO:0005509
CDK2	cyclin dependent kinase 2	PF3D7_1121300	PF11_0220	hypothetical protein	Troph/ Gametocytes	
CDK2	cyclin dependent kinase 2	PF3D7_1135100	PF11_0362	protein phosphatase, putative [Plasmodium falciparum 3D7]	Ring/Schizont	None
CDK2	cyclin dependent kinase 2	PF3D7_1247400	PFL2275c	FK506-binding protein (FKBP)-type peptidyl-propyl isomerase [Plasmodium falciparum 3D7]	Troph	GO:0006457
CDK2	cyclin dependent kinase 2	PF3D7_1342400	PF13_0232	casein kinase II beta chain [Plasmodium falciparum 3D7]	Troph	GO:0004674
CFTR	CF transmembrane conductance regulator	PF3D7_0302600	PFC0125w	ABC transporter, putative	Gametocytes	
CFTR	CF transmembrane conductance regulator	PF3D7_1339900	PF13_0218			
CFTR	CF transmembrane conductance regulator	PF3D7_1352100	PF13_0271	ABC transporter, (heavy metal transporter family) [Plasmodium falciparum 3D7]	Global	GO:0006810

CFTR	CF transmembrane conductance regulator	PF3D7_1426500	PF14_0244	ABC transporter, putative	Gametocytes	
CFTR	CF transmembrane conductance regulator	PF3D7_1447900	PF14_0455			
CIAO1	cytosolic iron-sulfur assembly component 1	PF3D7_0816000	PF08_0065	nucleolar preribosomal assembly protein, putative [Plasmodium falciparum 3D7]	Ring	None
CIAO1	cytosolic iron-sulfur assembly component 1	PF3D7_0909900	PFI0480w	helicase with Zn-finger motif, putative [Plasmodium falciparum 3D7]	Global	GO:0003676
CIAO1	cytosolic iron-sulfur assembly component 1	PF3D7_1036700	PF10_0359			
CIAO1	cytosolic iron-sulfur assembly component 1	PF3D7_1309300	MAL13P1.45	U4/U6 small nuclear ribonucleoprotein, putative [Plasmodium falciparum 3D7]	Global	GO:0003676
CIRBP	cold inducible RNA binding protein	PF3D7_0317200	PFC0755c	protein kinase, putative [Plasmodium falciparum 3D7]	Schizont	GO:0006468
CIRBP	cold inducible RNA binding protein	PF3D7_1323400	PF13_0132	60S ribosomal protein L23a, putative [Plasmodium falciparum 3D7]	Ring	GO:0006412
CIRBP	cold inducible RNA binding protein	PF3D7_1367100	MAL13P1.338	U1 small nuclear ribonucleoprotein, putative [Plasmodium falciparum 3D7]	Global	GO:0006396

COASY	Coenzyme A synthase	PF3D7_0520600	PFE1035c	BIS(5'-nucleosyl)-tetraphosphatase (Diadenosine tetraphosphatase), putative [Plasmodium falciparum 3D7]	Ring/Troph	GO:0004081
COASY	Coenzyme A synthase	PF3D7_1443700	PF14_0415	dephospho-CoA kinase, putative	Sporozoites/ Merozoites	
COPA	COPI coat complex subunit alpha	PF3D7_0606700	PFF0330w	coatomer alpha subunit, putative [Plasmodium falciparum 3D7]	Global	GO:0006886
COPA	COPI coat complex subunit alpha	PF3D7_1145100	PF11_0463	coat protein, gamma subunit, putative [Plasmodium falciparum 3D7]	Global	GO:0006886
CPNE1	copine 1	PF3D7_1243900	PFL2110c	conserved protein [Plasmodium falciparum 3D7]		
CPNE3	copine 3	PF3D7_1243900	PFL2110c	conserved protein [Plasmodium falciparum 3D7]		
CRKL	CRK like proto-oncogene, adaptor protein	PF3D7_0515300	PFE0765w	phosphatidylinositol 3-kinase, putative [Plasmodium falciparum 3D7]	Global	GO:0006897
CRKL	CRK like proto-oncogene, adaptor protein	PF3D7_1121300	PF11_0220	hypothetical protein	Troph/ Gametocytes	
CRKL	CRK like proto-oncogene, adaptor protein	PF3D7_1243900	PFL2110c	conserved protein [Plasmodium falciparum 3D7]		

CRK	CRK proto-oncogene, adaptor protein	PF3D7_0515300	PFE0765w	phosphatidylinositol 3-kinase, putative [Plasmodium falciparum 3D7]	Global	GO:0006897
CRK	CRK proto-oncogene, adaptor protein	PF3D7_1121300	PF11_0220	hypothetical protein	Troph/ Gametocytes	
CRK	CRK proto-oncogene, adaptor protein	PF3D7_1243900	PFL2110c	conserved protein [Plasmodium falciparum 3D7]		
CRNN	cornulin	PF3D7_1243900	PFL2110c	conserved protein [Plasmodium falciparum 3D7]		
CUL1	cullin 1	PF3D7_0629800	PFF1445c	cullin-like protein, putative [Plasmodium falciparum 3D7]	Unknown	GO:0000082
CUL4A	cullin 4A	PF3D7_0629800	PFF1445c	cullin-like protein, putative [Plasmodium falciparum 3D7]	Unknown	GO:0000082
CUL4B	cullin 4B	PF3D7_0629800	PFF1445c	cullin-like protein, putative [Plasmodium falciparum 3D7]	Unknown	GO:0000082
DBNL	drebrin like	PF3D7_0823300	PF08_0034	histone acetyltransferase GCN5, putative [Plasmodium falciparum 3D7]	Global	GO:0006355
DBNL	drebrin like	PF3D7_1121300	PF11_0220	hypothetical protein	Troph/ Gametocytes	
DDHD2	DDHD domain containing 2	PF3D7_0317200	PFC0755c	protein kinase, putative [Plasmodium falciparum 3D7]	Schizont	GO:0006468

DDI2	DNA damage inducible 1 homolog 2	PF3D7_0317200	PFC0755c	protein kinase, putative [Plasmodium falciparum 3D7]	Schizont	GO:0006468
DDX17	DEAD-box helicase 17	PF3D7_0215700	PF02_0148			
DDX17	DEAD-box helicase 17	PF3D7_0909900	PFI0480w	helicase with Zn-finger motif, putative [Plasmodium falciparum 3D7]	Global	GO:0003676
DDX17	DEAD-box helicase 17	PF3D7_1239500	PFL1915w			
DDX17	DEAD-box helicase 17	PF3D7_1302700	MAL13P1.14	ATP dependent DEAD-box helicase, putative [Plasmodium falciparum 3D7]	Global	GO:0003676
DDX17	DEAD-box helicase 17	PF3D7_1313400	PF13_0077	DEAD box helicase, putative	Gametocytes	
DDX17	DEAD-box helicase 17	PF3D7_1324500	MAL13P1.134			
DDX17	DEAD-box helicase 17	PF3D7_1408400	PF14_0081	DNA repair helicase, putative	Merozoites	
DDX27	DEAD-box helicase 27	PF3D7_0215700	PF02_0148			
DDX3X	DEAD-box helicase 3 X-linked	PF3D7_0215700	PF02_0148			
DDX3X	DEAD-box helicase 3 X-linked	PF3D7_0909900	PFI0480w	helicase with Zn-finger motif, putative [Plasmodium falciparum 3D7]	Global	GO:0003676
DDX3X	DEAD-box helicase 3 X-linked	PF3D7_1313400	PF13_0077	DEAD box helicase, putative	Gametocytes	
DGKA	diacylglycerol kinase alpha	PF3D7_0317200	PFC0755c	protein kinase, putative [Plasmodium falciparum 3D7]	Schizont	GO:0006468

DGKA	diacylglycerol kinase alpha	PF3D7_1243900	PFL2110c	conserved protein [Plasmodium falciparum 3D7]		
DHX15	DEAH-box helicase 15	PF3D7_0215700	PF02_0148			
DHX15	DEAH-box helicase 15	PF3D7_0909900	PFI0480w	helicase with Zn-finger motif, putative [Plasmodium falciparum 3D7]	Global	GO:0003676
DHX15	DEAH-box helicase 15	PF3D7_1313400	PF13_0077	DEAD box helicase, putative	Gametocytes	
DHX9	DExH-box helicase 9	PF3D7_0215700	PF02_0148			
DHX9	DExH-box helicase 9	PF3D7_0909900	PFI0480w	helicase with Zn-finger motif, putative [Plasmodium falciparum 3D7]	Global	GO:0003676
DHX9	DExH-box helicase 9	PF3D7_1239500	PFL1915w			
DHX9	DExH-box helicase 9	PF3D7_1302700	MAL13P1.14	ATP dependent DEAD-box helicase, putative [Plasmodium falciparum 3D7]	Global	GO:0003676
DHX9	DExH-box helicase 9	PF3D7_1313400	PF13_0077	DEAD box helicase, putative	Gametocytes	
DIP2B	disco interacting protein 2 homolog B	PF3D7_0627800	PFF1350c			
DOHH	deoxyhypusine hydroxylase	PF3D7_0317200	PFC0755c	protein kinase, putative [Plasmodium falciparum 3D7]	Schizont	GO:0006468
DOHH	deoxyhypusine hydroxylase	PF3D7_1338100	MAL13P1.190	proteasome regulatory component, putative [Plasmodium falciparum 3D7]	Global	GO:0006511

DPP9	dipeptidyl peptidase 9	PF3D7_0321500	PFC0950c	peptidase, putative [Plasmodium falciparum 3D7]	Troph	GO:0006508
DRG2	developmentally regulated GTP binding protein 2	PF3D7_0524400	PFE1215c	cytosolic preribosomal GTP-binding protein, putative [Plasmodium falciparum 3D7]	Ring	GO:0005525
DRG2	developmentally regulated GTP binding protein 2	PF3D7_0824300	MAL8P1.33			
DRG2	developmentally regulated GTP binding protein 2	PF3D7_1358900	MAL13P1.294	GTP binding protein, putative [Plasmodium falciparum 3D7]	Global	GO:0007165
EIF3F	eukaryotic translation initiation factor 3 subunit F	PF3D7_0612100	PFF0590c	homologue of human HSPC025 [Plasmodium falciparum 3D7]	Unknown	None
EIF3F	eukaryotic translation initiation factor 3 subunit F	PF3D7_1338100	MAL13P1.190	proteasome regulatory component, putative [Plasmodium falciparum 3D7]	Global	GO:0006511
EIF3G	eukaryotic translation initiation factor 3 subunit G	PF3D7_0317200	PFC0755c	protein kinase, putative [Plasmodium falciparum 3D7]	Schizont	GO:0006468
EIF3G	eukaryotic translation initiation factor 3 subunit G	PF3D7_1323400	PF13_0132	60S ribosomal protein L23a, putative [Plasmodium falciparum 3D7]	Ring	GO:0006412
EIF3G	eukaryotic translation initiation factor 3 subunit G	PF3D7_1329300	PF13_0149	chromatin assembly factor 1 subunit, putative [Plasmodium falciparum 3D7]	Global	GO:0006333

EIF3G	eukaryotic translation initiation factor 3 subunit G	PF3D7_1367100	MAL13P1.338	U1 small nuclear ribonucleoprotein, putative [Plasmodium falciparum 3D7]	Global	GO:0006396
EIF3I	eukaryotic translation initiation factor 3 subunit I	PF3D7_1036700	PF10_0359			
EIF3I	eukaryotic translation initiation factor 3 subunit I	PF3D7_1329300	PF13_0149	chromatin assembly factor 1 subunit, putative [Plasmodium falciparum 3D7]	Global	GO:0006333
EIF3K	eukaryotic translation initiation factor 3 subunit K	PF3D7_0612100	PFF0590c	homologue of human HSPC025 [Plasmodium falciparum 3D7]	Unknown	None
EIF3K	eukaryotic translation initiation factor 3 subunit K	PF3D7_1338100	MAL13P1.190	proteasome regulatory component, putative [Plasmodium falciparum 3D7]	Global	GO:0006511
EIF3M	eukaryotic translation initiation factor 3 subunit M	PF3D7_0612100	PFF0590c	homologue of human HSPC025 [Plasmodium falciparum 3D7]	Unknown	None
EIF3M	eukaryotic translation initiation factor 3 subunit M	PF3D7_1338100	MAL13P1.190	proteasome regulatory component, putative [Plasmodium falciparum 3D7]	Global	GO:0006511
FADD	Fas associated via death domain	PF3D7_0317200	PFC0755c	protein kinase, putative [Plasmodium falciparum 3D7]	Schizont	GO:0006468
FKBP2	FKBP prolyl isomerase 2	PF3D7_0317200	PFC0755c	protein kinase, putative [Plasmodium falciparum 3D7]	Schizont	GO:0006468

FKBP2	FKBP prolyl isomerase 2	PF3D7_1121 300	PF11_0220	hypothetical protein	Troph/ Gametocytes	
FKBP2	FKBP prolyl isomerase 2	PF3D7_1247 400	PFL2275c	FK506-binding protein (FKBP)-type peptidyl-propyl isomerase [Plasmodium falciparum 3D7]	Troph	GO:0006457
FKBP3	FKBP prolyl isomerase 3	PF3D7_0317 200	PFC0755c	protein kinase, putative [Plasmodium falciparum 3D7]	Schizont	GO:0006468
FKBP3	FKBP prolyl isomerase 3	PF3D7_1121 300	PF11_0220	hypothetical protein	Troph/ Gametocytes	
FKBP3	FKBP prolyl isomerase 3	PF3D7_1247 400	PFL2275c	FK506-binding protein (FKBP)-type peptidyl-propyl isomerase [Plasmodium falciparum 3D7]	Troph	GO:0006457
FKBP4	FKBP prolyl isomerase 4	PF3D7_0317 200	PFC0755c	protein kinase, putative [Plasmodium falciparum 3D7]	Schizont	GO:0006468
FKBP4	FKBP prolyl isomerase 4	PF3D7_1121 300	PF11_0220	hypothetical protein	Troph/ Gametocytes	
FKBP4	FKBP prolyl isomerase 4	PF3D7_1247 400	PFL2275c	FK506-binding protein (FKBP)-type peptidyl-propyl isomerase [Plasmodium falciparum 3D7]	Troph	GO:0006457
FKBP5	FKBP prolyl isomerase 5	PF3D7_0317 200	PFC0755c	protein kinase, putative [Plasmodium falciparum 3D7]	Schizont	GO:0006468
FKBP5	FKBP prolyl isomerase 5	PF3D7_1121 300	PF11_0220	hypothetical protein	Troph/ Gametocytes	

FKBP5	FKBP prolyl isomerase 5	PF3D7_1247 400	PFL2275c	FK506-binding protein (FKBP)-type peptidyl-propyl isomerase [Plasmodium falciparum 3D7]	Troph	GO:0006457
FLNA	filamin A	PF3D7_1121 300	PF11_0220	hypothetical protein	Troph/ Gametocytes	
FLNB	filamin B	PF3D7_1121 300	PF11_0220	hypothetical protein	Troph/ Gametocytes	
FLNC	filamin C	PF3D7_1121 300	PF11_0220	hypothetical protein	Troph/ Gametocytes	
FUBP1	far upstream element binding protein 1	PF3D7_0605 100	PFF0250w	RNA binding protein, putative [Plasmodium falciparum 3D7]	Global	GO:0003723
G3BP1	G3BP stress granule assembly factor 1	PF3D7_0317 200	PFC0755c	protein kinase, putative [Plasmodium falciparum 3D7]	Schizont	GO:0006468
G3BP2	G3BP stress granule assembly factor 2	PF3D7_0317 200	PFC0755c	protein kinase, putative [Plasmodium falciparum 3D7]	Schizont	GO:0006468
GAB1	GRB2 associated binding protein 1	PF3D7_0317 200	PFC0755c	protein kinase, putative [Plasmodium falciparum 3D7]	Schizont	GO:0006468
GAB1	GRB2 associated binding protein 1	PF3D7_1121 300	PF11_0220	hypothetical protein	Troph/ Gametocytes	
GAB1	GRB2 associated binding protein 1	PF3D7_1329 300	PF13_0149	chromatin assembly factor 1 subunit, putative [Plasmodium falciparum 3D7]	Global	GO:0006333
GAB1	GRB2 associated binding protein 1	PF3D7_1359 800	MAL13P1.297			

GAK	cyclin G associated kinase	PF3D7_1342400	PF13_0232	casein kinase II beta chain [Plasmodium falciparum 3D7]	Troph	GO:0004674
GGA3	golgi associated, gamma adaptin ear containing, ARF binding protein 3	PF3D7_1359800	MAL13P1.297			
GLRX3	glutaredoxin 3	PF3D7_1411400	PF14_0112			
GMDS	GDP-mannose 4,6-dehydratase	PF3D7_0813800	PF08_0077			
GMFB	glia maturation factor beta	PF3D7_0823300	PF08_0034	histone acetyltransferase GCN5, putative [Plasmodium falciparum 3D7]	Global	GO:0006355
GMFB	glia maturation factor beta	PF3D7_1121300	PF11_0220	hypothetical protein	Troph/ Gametocytes	
GMFG	glia maturation factor gamma	PF3D7_0823300	PF08_0034	histone acetyltransferase GCN5, putative [Plasmodium falciparum 3D7]	Global	GO:0006355
GMFG	glia maturation factor gamma	PF3D7_1121300	PF11_0220	hypothetical protein	Troph/ Gametocytes	
GMPPA	GDP-mannose pyrophosphorylase A	PF3D7_0828500	PF08_0009	translation initiation factor EIF-2b alpha subunit, putative [Plasmodium falciparum 3D7]	Global	GO:0006413
GMPPB	GDP-mannose pyrophosphorylase B	PF3D7_0828500	PF08_0009	translation initiation factor EIF-2b alpha subunit, putative [Plasmodium falciparum 3D7]	Global	GO:0006413

GNA13	G protein subunit alpha 13	PF3D7_1329300	PF13_0149	chromatin assembly factor 1 subunit, putative [Plasmodium falciparum 3D7]	Global	GO:0006333
GNA14	G protein subunit alpha 14	PF3D7_1329300	PF13_0149	chromatin assembly factor 1 subunit, putative [Plasmodium falciparum 3D7]	Global	GO:0006333
GNAI3	G protein subunit alpha i3	PF3D7_1329300	PF13_0149	chromatin assembly factor 1 subunit, putative [Plasmodium falciparum 3D7]	Global	GO:0006333
GNAQ	G protein subunit alpha q	PF3D7_1329300	PF13_0149	chromatin assembly factor 1 subunit, putative [Plasmodium falciparum 3D7]	Global	GO:0006333
GSTT1	glutathione S-transferase theta 1	PF3D7_1338300	PF13_0214	elongation factor 1-gamma, putative [Plasmodium falciparum 3D7]	Troph	GO:0006414
HACE1	HECT domain and ankyrin repeat containing E3 ubiquitin protein ligase 1	PF3D7_0309000	PFC0380w	dual-specificity protein phosphatase, putative	Troph/ Gametocytes	
HACE1	HECT domain and ankyrin repeat containing E3 ubiquitin protein ligase 1	PF3D7_0317200	PFC0755c	protein kinase, putative [Plasmodium falciparum 3D7]	Schizont	GO:0006468

HACE1	HECT domain and ankyrin repeat containing E3 ubiquitin protein ligase 1	PF3D7_0528500	PFE1420w			
HACE1	HECT domain and ankyrin repeat containing E3 ubiquitin protein ligase 1	PF3D7_1247800	PFL2290w	preprocathepsin c precursor, putative	Troph/ Gametocytes	
HACE1	HECT domain and ankyrin repeat containing E3 ubiquitin protein ligase 1	PF3D7_1359800	MAL13P1.297			
HBB	hemoglobin subunit beta	PF3D7_0623200	PFF1115w			
HBD	hypophosphatemic bone disease	PF3D7_0623200	PFF1115w			
HBG1	hemoglobin subunit gamma 1	PF3D7_0623200	PFF1115w			
HBM	hemoglobin subunit mu	PF3D7_0623200	PFF1115w			
HBS1L	HBS1 like translational GTPase	PF3D7_0212300	PF02_0115			
HBS1L	HBS1 like translational GTPase	PF3D7_0516600	PFE0830c	unknown protein, mb2	Merozoites	
HBS1L	HBS1 like translational GTPase	PF3D7_0524400	PFE1215c	cytosolic preribosomal GTP-binding protein, putative [Plasmodium falciparum 3D7]	Ring	GO:0005525
HBS1L	HBS1 like translational GTPase	PF3D7_0602400	PFF0115c			

HBS1L	HBS1 like translational GTPase	PF3D7_1123400	PF11_0245	translation elongation factor EF-1, subunit alpha, putative [Plasmodium falciparum 3D7]	Ring/Troph	GO:0006414
HBS1L	HBS1 like translational GTPase	PF3D7_1233000	PFL1590c	elongation factor G, putative [Plasmodium falciparum 3D7]	Troph	GO:0006414
HBS1L	HBS1 like translational GTPase	PF3D7_1358900	MAL13P1.294	GTP binding protein, putative [Plasmodium falciparum 3D7]	Global	GO:0007165
HBS1L	HBS1 like translational GTPase	PF3D7_1410600	PF14_0104	eukaryotic translation initiation factor 2 gamma subunit, putative [Plasmodium falciparum 3D7]	Global	GO:0006413
HCLS1	hematopoietic cell-specific Lyn substrate 1	PF3D7_1121300	PF11_0220	hypothetical protein	Troph/ Gametocytes	
HDAC6	histone deacetylase 6	PF3D7_0602400	PFF0115c			
HELLS	helicase, lymphoid specific	PF3D7_0215700	PF02_0148			
HELLS	helicase, lymphoid specific	PF3D7_1023900	PF10_0232	Chromodomain-helicase-DNA-binding protein 1 homolog, putative [Plasmodium falciparum 3D7]	Global	GO:0006333
HELLS	helicase, lymphoid specific	PF3D7_1302700	MAL13P1.14	ATP dependent DEAD-box helicase, putative [Plasmodium falciparum 3D7]	Global	GO:0003676

HELLS	helicase, lymphoid specific	PF3D7_1313400	PF13_0077	DEAD box helicase, putative	Gametocytes	
HNRPD		PF3D7_0317200	PFC0755c	protein kinase, putative [Plasmodium falciparum 3D7]	Schizont	GO:0006468
HNRPD		PF3D7_1367100	MAL13P1.338	U1 small nuclear ribonucleoprotein, putative [Plasmodium falciparum 3D7]	Global	GO:0006396
HNRPK		PF3D7_0605100	PFF0250w	RNA binding protein, putative [Plasmodium falciparum 3D7]	Global	GO:0003723
HSPB1	heat shock protein family B (small) member 1	PF3D7_0816500	MAL8P1.78	small heat shock protein	Merozoites	
HUWE1	HECT, UBA and WWE domain containing E3 ubiquitin protein ligase 1	PF3D7_0317200	PFC0755c	protein kinase, putative [Plasmodium falciparum 3D7]	Schizont	GO:0006468
ICAM4	intercellular adhesion molecule 4 (Landsteiner-Wiener blood group)	PF3D7_0616500	PFF0800w			
IFIT5	interferon induced protein with tetratricopeptide repeats 5	PF3D7_0631000	PFF1505w	TPR-like domain containing protein, putative [Plasmodium falciparum 3D7]		
IGHG1	immunoglobulin heavy constant gamma 1 (G1m marker)	PF3D7_0616500	PFF0800w			
IGHM	immunoglobulin heavy constant mu	PF3D7_0616500	PFF0800w			

IRAK4	interleukin 1 receptor associated kinase 4	PF3D7_0317200	PFC0755c	protein kinase, putative [Plasmodium falciparum 3D7]	Schizont	GO:0006468
IRAK4	interleukin 1 receptor associated kinase 4	PF3D7_1121300	PF11_0220	hypothetical protein	Troph/ Gametocytes	
IRAK4	interleukin 1 receptor associated kinase 4	PF3D7_1247400	PFL2275c	FK506-binding protein (FKBP)-type peptidyl-propyl isomerase [Plasmodium falciparum 3D7]	Troph	GO:0006457
ITSN1	intersectin 1	PF3D7_1121300	PF11_0220	hypothetical protein	Troph/ Gametocytes	
ITSN1	intersectin 1	PF3D7_1243900	PFL2110c	conserved protein [Plasmodium falciparum 3D7]		
KALRN	kalirin RhoGEF kinase	PF3D7_0317200	PFC0755c	protein kinase, putative [Plasmodium falciparum 3D7]	Schizont	GO:0006468
KALRN	kalirin RhoGEF kinase	PF3D7_1135100	PF11_0362	protein phosphatase, putative [Plasmodium falciparum 3D7]	Ring/Schizont	None
KALRN	kalirin RhoGEF kinase	PF3D7_1247400	PFL2275c	FK506-binding protein (FKBP)-type peptidyl-propyl isomerase [Plasmodium falciparum 3D7]	Troph	GO:0006457
KALRN	kalirin RhoGEF kinase	PF3D7_1359800	MAL13P1.297			
KCTD7	potassium channel tetramerization domain containing 7	PF3D7_1238700	PFL1875w	conserved Plasmodium protein [Plasmodium falciparum 3D7]	Unknown	GO:0006813

LASP1	LIM and SH3 protein 1	PF3D7_1121300	PF11_0220	hypothetical protein	Troph/ Gametocytes	
LDHA	lactate dehydrogenase A	PF3D7_1325200	PF13_0144	oxidoreductase, putative	Merozoites/ Gametocytes	
LDHB	lactate dehydrogenase B	PF3D7_1325200	PF13_0144	oxidoreductase, putative	Merozoites/ Gametocytes	
LSM1	LSM1 homolog, mRNA degradation associated	PF3D7_1309300	MAL13P1.45	U4/U6 small nuclear ribonucleoprotein, putative [Plasmodium falciparum 3D7]	Global	GO:0003676
LSM1	LSM1 homolog, mRNA degradation associated	PF3D7_1367100	MAL13P1.338	U1 small nuclear ribonucleoprotein, putative [Plasmodium falciparum 3D7]	Global	GO:0006396
LSM2	LSM2 homolog, U6 small nuclear RNA and mRNA degradation associated	PF3D7_1309300	MAL13P1.45	U4/U6 small nuclear ribonucleoprotein, putative [Plasmodium falciparum 3D7]	Global	GO:0003676
LSM2	LSM2 homolog, U6 small nuclear RNA and mRNA degradation associated	PF3D7_1367100	MAL13P1.338	U1 small nuclear ribonucleoprotein, putative [Plasmodium falciparum 3D7]	Global	GO:0006396
LSM3	LSM3 homolog, U6 small nuclear RNA and mRNA degradation associated	PF3D7_1309300	MAL13P1.45	U4/U6 small nuclear ribonucleoprotein, putative [Plasmodium falciparum 3D7]	Global	GO:0003676
LSM4	LSM4 homolog, U6 small nuclear RNA and mRNA degradation associated	PF3D7_1309300	MAL13P1.45	U4/U6 small nuclear ribonucleoprotein, putative [Plasmodium falciparum 3D7]	Global	GO:0003676
LSM4	LSM4 homolog, U6 small nuclear RNA and mRNA degradation associated	PF3D7_1367100	MAL13P1.338	U1 small nuclear ribonucleoprotein, putative [Plasmodium falciparum 3D7]	Global	GO:0006396

LSM5	LSM5 homolog, U6 small nuclear RNA and mRNA degradation associated	PF3D7_1367100	MAL13P1.338	U1 small nuclear ribonucleoprotein, putative [Plasmodium falciparum 3D7]	Global	GO:0006396
LSM6	LSM6 homolog, U6 small nuclear RNA and mRNA degradation associated	PF3D7_1309300	MAL13P1.45	U4/U6 small nuclear ribonucleoprotein, putative [Plasmodium falciparum 3D7]	Global	GO:0003676
LSM6	LSM6 homolog, U6 small nuclear RNA and mRNA degradation associated	PF3D7_1367100	MAL13P1.338	U1 small nuclear ribonucleoprotein, putative [Plasmodium falciparum 3D7]	Global	GO:0006396
LSM7	LSM7 homolog, U6 small nuclear RNA and mRNA degradation associated	PF3D7_1367100	MAL13P1.338	U1 small nuclear ribonucleoprotein, putative [Plasmodium falciparum 3D7]	Global	GO:0006396
LSM8	LSM8 homolog, U6 small nuclear RNA associated	PF3D7_1309300	MAL13P1.45	U4/U6 small nuclear ribonucleoprotein, putative [Plasmodium falciparum 3D7]	Global	GO:0003676
LSM8	LSM8 homolog, U6 small nuclear RNA associated	PF3D7_1367100	MAL13P1.338	U1 small nuclear ribonucleoprotein, putative [Plasmodium falciparum 3D7]	Global	GO:0006396
MARK3	microtubule affinity regulating kinase 3	PF3D7_0309000	PFC0380w	dual-specificity protein phosphatase, putative	Troph/ Gametocytes	
MARK3	microtubule affinity regulating kinase 3	PF3D7_0317200	PFC0755c	protein kinase, putative [Plasmodium falciparum 3D7]	Schizont	GO:0006468
MARK3	microtubule affinity regulating kinase 3	PF3D7_1121300	PF11_0220	hypothetical protein	Troph/ Gametocytes	

MARK3	microtubule affinity regulating kinase 3	PF3D7_1135 100	PF11_0362	protein phosphatase , putative [Plasmodium falciparum 3D7]	Ring/Schizont	None
MARK3	microtubule affinity regulating kinase 3	PF3D7_1247 400	PFL2275c	FK506-binding protein (FKBP)-type peptidyl-propyl isomerase [Plasmodium falciparum 3D7]	Troph	GO:0006457
MARK3	microtubule affinity regulating kinase 3	PF3D7_1342 400	PF13_0232	casein kinase II beta chain [Plasmodium falciparum 3D7]	Troph	GO:0004674
MERTK	MER proto-oncogene, tyrosine kinase	PF3D7_0309 000	PFC0380w	dual-specificity protein phosphatase , putative	Troph/ Gametocytes	
MERTK	MER proto-oncogene, tyrosine kinase	PF3D7_0317 200	PFC0755c	protein kinase, putative [Plasmodium falciparum 3D7]	Schizont	GO:0006468
MERTK	MER proto-oncogene, tyrosine kinase	PF3D7_1121 300	PF11_0220	hypothetical protein	Troph/ Gametocytes	
MERTK	MER proto-oncogene, tyrosine kinase	PF3D7_1247 400	PFL2275c	FK506-binding protein (FKBP)-type peptidyl-propyl isomerase [Plasmodium falciparum 3D7]	Troph	GO:0006457
MERTK	MER proto-oncogene, tyrosine kinase	PF3D7_1329 300	PF13_0149	chromatin assembly factor 1 subunit, putative [Plasmodium falciparum 3D7]	Global	GO:0006333

MIB1	mindbomb E3 ubiquitin protein ligase 1	PF3D7_0309000	PFC0380w	dual-specificity protein phosphatase, putative	Troph/ Gametocytes	
MIB1	mindbomb E3 ubiquitin protein ligase 1	PF3D7_0317200	PFC0755c	protein kinase, putative [Plasmodium falciparum 3D7]	Schizont	GO:0006468
MIB1	mindbomb E3 ubiquitin protein ligase 1	PF3D7_0528500	PFE1420w			
MIB1	mindbomb E3 ubiquitin protein ligase 1	PF3D7_1247800	PFL2290w	preprocathepsin c precursor, putative	Troph/ Gametocytes	
MIB1	mindbomb E3 ubiquitin protein ligase 1	PF3D7_1358000	MAL13P1.285	patatin-like phospholipase, putative [Plasmodium falciparum 3D7]	Global	GO:0006629
MIB1	mindbomb E3 ubiquitin protein ligase 1	PF3D7_1359800	MAL13P1.297			
MON2	MON2 homolog, regulator of endosome-to-Golgi trafficking	PF3D7_1359800	MAL13P1.297			
MOS	MOS proto-oncogene, serine/threonine kinase	PF3D7_0309000	PFC0380w	dual-specificity protein phosphatase, putative	Troph/ Gametocytes	
MOS	MOS proto-oncogene, serine/threonine kinase	PF3D7_0317200	PFC0755c	protein kinase, putative [Plasmodium falciparum 3D7]	Schizont	GO:0006468
MOS	MOS proto-oncogene, serine/threonine kinase	PF3D7_1121300	PF11_0220	hypothetical protein	Troph/ Gametocytes	
MOS	MOS proto-oncogene, serine/threonine kinase	PF3D7_1135100	PF11_0362	protein phosphatase, putative [Plasmodium falciparum 3D7]	Ring/Schizont	None

MOS	MOS proto-oncogene, serine/threonine kinase	PF3D7_1247400	PFL2275c	FK506-binding protein (FKBP)-type peptidyl-propyl isomerase [Plasmodium falciparum 3D7]	Troph	GO:0006457
MOS	MOS proto-oncogene, serine/threonine kinase	PF3D7_1342400	PF13_0232	casein kinase II beta chain [Plasmodium falciparum 3D7]	Troph	GO:0004674
MTOR	mechanistic target of rapamycin kinase	PF3D7_0515300	PFE0765w	phosphatidylinositol 3-kinase, putative [Plasmodium falciparum 3D7]	Global	GO:0006897
MTOR	mechanistic target of rapamycin kinase	PF3D7_1247400	PFL2275c	FK506-binding protein (FKBP)-type peptidyl-propyl isomerase [Plasmodium falciparum 3D7]	Troph	GO:0006457
MTOR	mechanistic target of rapamycin kinase	PF3D7_1329300	PF13_0149	chromatin assembly factor 1 subunit, putative [Plasmodium falciparum 3D7]	Global	GO:0006333
MTPN	myotrophin	PF3D7_0309000	PFC0380w	dual-specificity protein phosphatase, putative	Troph/ Gametocytes	
MTPN	myotrophin	PF3D7_0317200	PFC0755c	protein kinase, putative [Plasmodium falciparum 3D7]	Schizont	GO:0006468
MTPN	myotrophin	PF3D7_0528500	PFE1420w			
MTPN	myotrophin	PF3D7_1247800	PFL2290w	preprocathepsin c precursor, putative	Troph/ Gametocytes	

MYL6	myosin light chain 6	PF3D7_0317200	PFC0755c	protein kinase, putative [Plasmodium falciparum 3D7]	Schizont	GO:0006468
MYLK2	myosin light chain kinase 2	PF3D7_0309000	PFC0380w	dual-specificity protein phosphatase, putative	Troph/ Gametocytes	
MYLK2	myosin light chain kinase 2	PF3D7_0317200	PFC0755c	protein kinase, putative [Plasmodium falciparum 3D7]	Schizont	GO:0006468
MYLK2	myosin light chain kinase 2	PF3D7_1030800	PF10_0301	calmodulin, putative [Plasmodium falciparum 3D7]	Global	GO:0005509
MYLK2	myosin light chain kinase 2	PF3D7_1121300	PF11_0220	hypothetical protein	Troph/ Gametocytes	
MYLK2	myosin light chain kinase 2	PF3D7_1247400	PFL2275c	FK506-binding protein (FKBP)-type peptidyl-propyl isomerase [Plasmodium falciparum 3D7]	Troph	GO:0006457
MYLK2	myosin light chain kinase 2	PF3D7_1342400	PF13_0232	casein kinase II beta chain [Plasmodium falciparum 3D7]	Troph	GO:0004674
MYO1D	myosin ID	PF3D7_1030800	PF10_0301	calmodulin, putative [Plasmodium falciparum 3D7]	Global	GO:0005509
NCBP1	nuclear cap binding protein subunit 1	PF3D7_1313400	PF13_0077	DEAD box helicase, putative	Gametocytes	
NCBP2	nuclear cap binding protein subunit 2	PF3D7_0317200	PFC0755c	protein kinase, putative [Plasmodium falciparum 3D7]	Schizont	GO:0006468

NCBP2	nuclear cap binding protein subunit 2	PF3D7_1323400	PF13_0132	60S ribosomal protein L23a, putative [Plasmodium falciparum 3D7]	Ring	GO:0006412
NCBP2	nuclear cap binding protein subunit 2	PF3D7_1367100	MAL13P1.338	U1 small nuclear ribonucleoprotein, putative [Plasmodium falciparum 3D7]	Global	GO:0006396
NOS1	nitric oxide synthase 1	PF3D7_0623200	PFF1115w			
NOS1	nitric oxide synthase 1	PF3D7_0812200	MAL8P1.99	GTPase, putative [Plasmodium falciparum 3D7]	Global	GO:0006508
NOS1	nitric oxide synthase 1	PF3D7_1011900	PF10_0116			
NUDT4	nudix hydrolase 4	PF3D7_0520600	PFE1035c	BIS(5'-nucleosyl)-tetraphosphatase (Diadenosine tetraphosphatase), putative [Plasmodium falciparum 3D7]	Ring/Troph	GO:0004081
NUDT5	nudix hydrolase 5	PF3D7_0520600	PFE1035c	BIS(5'-nucleosyl)-tetraphosphatase (Diadenosine tetraphosphatase), putative [Plasmodium falciparum 3D7]	Ring/Troph	GO:0004081
NUP43	nucleoporin 43	PF3D7_1122900	PF11_0240	dynein heavy chain, putative	Merozoites/ Gametocytes	
NUP43	nucleoporin 43	PF3D7_1329300	PF13_0149	chromatin assembly factor 1 subunit, putative [Plasmodium falciparum 3D7]	Global	GO:0006333

OLA1	Obg like ATPase 1	PF3D7_0524400	PFE1215c	cytosolic preribosomal GTP-binding protein, putative [Plasmodium falciparum 3D7]	Ring	GO:0005525
OLA1	Obg like ATPase 1	PF3D7_0824300	MAL8P1.33			
OLA1	Obg like ATPase 1	PF3D7_1358900	MAL13P1.294	GTP binding protein, putative [Plasmodium falciparum 3D7]	Global	GO:0007165
OSBP2	oxysterol binding protein 2	PF3D7_0317200	PFC0755c	protein kinase, putative [Plasmodium falciparum 3D7]	Schizont	GO:0006468
OSBP2	oxysterol binding protein 2	PF3D7_1121300	PF11_0220	hypothetical protein	Troph/ Gametocytes	
OSBP2	oxysterol binding protein 2	PF3D7_1329300	PF13_0149	chromatin assembly factor 1 subunit, putative [Plasmodium falciparum 3D7]	Global	GO:0006333
OSBP2	oxysterol binding protein 2	PF3D7_1359800	MAL13P1.297			
OSTF1	osteoclast stimulating factor 1	PF3D7_0309000	PFC0380w	dual-specificity protein phosphatase, putative	Troph/ Gametocytes	
OSTF1	osteoclast stimulating factor 1	PF3D7_0317200	PFC0755c	protein kinase, putative [Plasmodium falciparum 3D7]	Schizont	GO:0006468
OSTF1	osteoclast stimulating factor 1	PF3D7_0528500	PFE1420w			
OSTF1	osteoclast stimulating factor 1	PF3D7_1121300	PF11_0220	hypothetical protein	Troph/ Gametocytes	
OSTF1	osteoclast stimulating factor 1	PF3D7_1247800	PFL2290w	preprocathepsin c precursor, putative	Troph/ Gametocytes	

OSTF1	osteoclast stimulating factor 1	PF3D7_1359800	MAL13P1.297			
OXSR1	oxidative stress responsive kinase 1	PF3D7_0309000	PFC0380w	dual-specificity protein phosphatase, putative	Troph/ Gametocytes	
OXSR1	oxidative stress responsive kinase 1	PF3D7_0317200	PFC0755c	protein kinase, putative [Plasmodium falciparum 3D7]	Schizont	GO:0006468
OXSR1	oxidative stress responsive kinase 1	PF3D7_1030800	PF10_0301	calmodulin, putative [Plasmodium falciparum 3D7]	Global	GO:0005509
OXSR1	oxidative stress responsive kinase 1	PF3D7_1121300	PF11_0220	hypothetical protein	Troph/ Gametocytes	
OXSR1	oxidative stress responsive kinase 1	PF3D7_1135100	PF11_0362	protein phosphatase, putative [Plasmodium falciparum 3D7]	Ring/Schizont	None
OXSR1	oxidative stress responsive kinase 1	PF3D7_1247400	PFL2275c	FK506-binding protein (FKBP)-type peptidyl-propyl isomerase [Plasmodium falciparum 3D7]	Troph	GO:0006457
OXSR1	oxidative stress responsive kinase 1	PF3D7_1342400	PF13_0232	casein kinase II beta chain [Plasmodium falciparum 3D7]	Troph	GO:0004674
PAAF1	proteasomal ATPase associated factor 1	PF3D7_0816000	PF08_0065	nucleolar preribosomal assembly protein, putative [Plasmodium falciparum 3D7]	Ring	None

PAAF1	proteasomal ATPase associated factor 1	PF3D7_0909900	PFI0480w	helicase with Zn-finger motif, putative [Plasmodium falciparum 3D7]	Global	GO:0003676
PAIP1	poly(A) binding protein interacting protein 1	PF3D7_1313400	PF13_0077	DEAD box helicase, putative	Gametocytes	
PAK2	p21 (RAC1) activated kinase 2	PF3D7_0309000	PFC0380w	dual-specificity protein phosphatase, putative	Troph/ Gametocytes	
PAK2	p21 (RAC1) activated kinase 2	PF3D7_0317200	PFC0755c	protein kinase, putative [Plasmodium falciparum 3D7]	Schizont	GO:0006468
PAK2	p21 (RAC1) activated kinase 2	PF3D7_1030800	PF10_0301	calmodulin, putative [Plasmodium falciparum 3D7]	Global	GO:0005509
PAK2	p21 (RAC1) activated kinase 2	PF3D7_1121300	PF11_0220	hypothetical protein	Troph/ Gametocytes	
PAK2	p21 (RAC1) activated kinase 2	PF3D7_1135100	PF11_0362	protein phosphatase, putative [Plasmodium falciparum 3D7]	Ring/Schizont	None
PAK2	p21 (RAC1) activated kinase 2	PF3D7_1247400	PFL2275c	FK506-binding protein (FKBP)-type peptidyl-propyl isomerase [Plasmodium falciparum 3D7]	Troph	GO:0006457
PAK2	p21 (RAC1) activated kinase 2	PF3D7_1342400	PF13_0232	casein kinase II beta chain [Plasmodium falciparum 3D7]	Troph	GO:0004674

PCBP1	poly(rC) binding protein 1	PF3D7_0605 100	PFF0250w	RNA binding protein, putative [Plasmodium falciparum 3D7]	Global	GO:0003723
PCBP2	poly(rC) binding protein 2	PF3D7_0605 100	PFF0250w	RNA binding protein, putative [Plasmodium falciparum 3D7]	Global	GO:0003723
PCCA	propionyl-CoA carboxylase subunit alpha	PF3D7_1308 200	PF13_0044	carbamoyl phosphate synthetase [Plasmodium falciparum 3D7]	Ring/Troph	GO:0006807
PDCD6	programmed cell death 6	PF3D7_0317 200	PFC0755c	protein kinase, putative [Plasmodium falciparum 3D7]	Schizont	GO:0006468
PDCD6	programmed cell death 6	PF3D7_1030 800	PF10_0301	calmodulin, putative [Plasmodium falciparum 3D7]	Global	GO:0005509
PDCD6	programmed cell death 6	PF3D7_1247 400	PFL2275c	FK506-binding protein (FKBP)-type peptidyl-propyl isomerase [Plasmodium falciparum 3D7]	Troph	GO:0006457
PDCL3	phosducin like 3	PF3D7_1329 300	PF13_0149	chromatin assembly factor 1 subunit, putative [Plasmodium falciparum 3D7]	Global	GO:0006333
PDE6D	phosphodiesterase 6D	PF3D7_1359 800	MAL13P1.297			
PDIA2	protein disulfide isomerase family A member 2	PF3D7_1411 400	PF14_0112			

PDIA3	protein disulfide isomerase family A member 3	PF3D7_1411400	PF14_0112			
PEA15	proliferation and apoptosis adaptor protein 15	PF3D7_0317200	PFC0755c	protein kinase, putative [Plasmodium falciparum 3D7]	Schizont	GO:0006468
PEBP1	phosphatidylethanolamine binding protein 1	PF3D7_1424100	PF14_0230	60S ribosomal protein L5, putative [Plasmodium falciparum 3D7]	Ring/Troph	GO:0006412
PEF1	penta-EF-hand domain containing 1	PF3D7_0317200	PFC0755c	protein kinase, putative [Plasmodium falciparum 3D7]	Schizont	GO:0006468
PEF1	penta-EF-hand domain containing 1	PF3D7_1247400	PFL2275c	FK506-binding protein (FKBP)-type peptidyl-propyl isomerase [Plasmodium falciparum 3D7]	Troph	GO:0006457
PELO	pelota mRNA surveillance and ribosome rescue factor	PF3D7_0212300	PF02_0115			
PELO	pelota mRNA surveillance and ribosome rescue factor	PF3D7_1123400	PF11_0245	translation elongation factor EF-1, subunit alpha, putative [Plasmodium falciparum 3D7]	Ring/Troph	GO:0006414
PHKG2	phosphorylase kinase catalytic subunit gamma 2	PF3D7_0309000	PFC0380w	dual-specificity protein phosphatase, putative	Troph/ Gametocytes	

PHKG2	phosphorylase kinase catalytic subunit gamma 2	PF3D7_0317200	PFC0755c	protein kinase, putative [Plasmodium falciparum 3D7]	Schizont	GO:0006468
PHKG2	phosphorylase kinase catalytic subunit gamma 2	PF3D7_1030800	PF10_0301	calmodulin, putative [Plasmodium falciparum 3D7]	Global	GO:0005509
PHKG2	phosphorylase kinase catalytic subunit gamma 2	PF3D7_1121300	PF11_0220	hypothetical protein	Troph/ Gametocytes	
PHKG2	phosphorylase kinase catalytic subunit gamma 2	PF3D7_1135100	PF11_0362	protein phosphatase, putative [Plasmodium falciparum 3D7]	Ring/Schizont	None
PHKG2	phosphorylase kinase catalytic subunit gamma 2	PF3D7_1216900	PFL0815w	DNA-binding chaperone, putative [Plasmodium falciparum 3D7]	Ring/Troph	GO:0003677
PHKG2	phosphorylase kinase catalytic subunit gamma 2	PF3D7_1247400	PFL2275c	FK506-binding protein (FKBP)-type peptidyl-propyl isomerase [Plasmodium falciparum 3D7]	Troph	GO:0006457
PHKG2	phosphorylase kinase catalytic subunit gamma 2	PF3D7_1342400	PF13_0232	casein kinase II beta chain [Plasmodium falciparum 3D7]	Troph	GO:0004674
PKN1	protein kinase N1	PF3D7_0309000	PFC0380w	dual-specificity protein phosphatase, putative	Troph/ Gametocytes	
PKN1	protein kinase N1	PF3D7_0317200	PFC0755c	protein kinase, putative [Plasmodium falciparum 3D7]	Schizont	GO:0006468

PKN1	protein kinase N1	PF3D7_1030800	PF10_0301	calmodulin, putative [Plasmodium falciparum 3D7]	Global	GO:0005509
PKN1	protein kinase N1	PF3D7_1121300	PF11_0220	hypothetical protein	Troph/ Gametocytes	
PKN1	protein kinase N1	PF3D7_1135100	PF11_0362	protein phosphatase, putative [Plasmodium falciparum 3D7]	Ring/Schizont	None
PKN1	protein kinase N1	PF3D7_1247400	PFL2275c	FK506-binding protein (FKBP)-type peptidyl-propyl isomerase [Plasmodium falciparum 3D7]	Troph	GO:0006457
PKN1	protein kinase N1	PF3D7_1342400	PF13_0232	casein kinase II beta chain [Plasmodium falciparum 3D7]	Troph	GO:0004674
PLCB3	phospholipase C beta 3	PF3D7_1243900	PFL2110c	conserved protein [Plasmodium falciparum 3D7]		
PLEK2	pleckstrin 2	PF3D7_0317200	PFC0755c	protein kinase, putative [Plasmodium falciparum 3D7]	Schizont	GO:0006468
PLEK2	pleckstrin 2	PF3D7_1121300	PF11_0220	hypothetical protein	Troph/ Gametocytes	
PLEK2	pleckstrin 2	PF3D7_1359800	MAL13P1.297			
PPCS	phosphopantothenoylcysteine synthetase	PF3D7_0412300	PFD0610w	phosphopantothenoylcysteine synthetase, putative [Plasmodium falciparum 3D7]	Global	None
PPIL4	peptidylprolyl isomerase like 4	PF3D7_0317200	PFC0755c	protein kinase, putative [Plasmodium falciparum 3D7]	Schizont	GO:0006468

PPM1A	protein phosphatase, Mg2+/Mn2+ dependent 1A	PF3D7_0317200	PFC0755c	protein kinase, putative [Plasmodium falciparum 3D7]	Schizont	GO:0006468
PPM1A	protein phosphatase, Mg2+/Mn2+ dependent 1A	PF3D7_1135100	PF11_0362	protein phosphatase, putative [Plasmodium falciparum 3D7]	Ring/Schizont	None
PPM1B	protein phosphatase, Mg2+/Mn2+ dependent 1B	PF3D7_0317200	PFC0755c	protein kinase, putative [Plasmodium falciparum 3D7]	Schizont	GO:0006468
PPM1B	protein phosphatase, Mg2+/Mn2+ dependent 1B	PF3D7_1135100	PF11_0362	protein phosphatase, putative [Plasmodium falciparum 3D7]	Ring/Schizont	None
PPME1	protein phosphatase methylesterase 1	PF3D7_0709700	PF07_0040	lysophospholipase, putative [Plasmodium falciparum 3D7]	Schizont	GO:0006644
PPME1	protein phosphatase methylesterase 1	PF3D7_1038900	PF10_0379	phospholipase, putative [Plasmodium falciparum 3D7]	Global	GO:0016787
PPME1	protein phosphatase methylesterase 1	PF3D7_1401500	PF14_0017			
PRDX5	peroxiredoxin 5	PF3D7_1430900	PF14_0288	cytochrome C oxidase subunit II precursor, putative	Gametocytes	
PRG2	proteoglycan 2, pro eosinophil major basic protein	PF3D7_0616500	PFF0800w			
PROZ	protein Z, vitamin K dependent plasma glycoprotein	PF3D7_0616500	PFF0800w			

PROZ	protein Z, vitamin K dependent plasma glycoprotein	PF3D7_0911300	PFI0550w	hypothetical protein	Sporozoites/ Merozoites	
PRPS1	phosphoribosyl pyrophosphate synthetase 1	PF3D7_1327800	PF13_0157	ribose-phosphate pyrophosphokinase, putative [Plasmodium falciparum 3D7]	Global	GO:0000105
PSMD1	proteasome 26S subunit, non-ATPase 1	PF3D7_0317200	PFC0755c	protein kinase, putative [Plasmodium falciparum 3D7]	Schizont	GO:0006468
PSMD1	proteasome 26S subunit, non-ATPase 1	PF3D7_1338100	MAL13P1.190	proteasome regulatory component, putative [Plasmodium falciparum 3D7]	Global	GO:0006511
PSMD3	proteasome 26S subunit, non-ATPase 3	PF3D7_1338100	MAL13P1.190	proteasome regulatory component, putative [Plasmodium falciparum 3D7]	Global	GO:0006511
PSMD6	proteasome 26S subunit, non-ATPase 6	PF3D7_1338100	MAL13P1.190	proteasome regulatory component, putative [Plasmodium falciparum 3D7]	Global	GO:0006511
PSMD7	proteasome 26S subunit, non-ATPase 7	PF3D7_0612100	PFF0590c	homologue of human HSPC025 [Plasmodium falciparum 3D7]	Unknown	None
PSMD7	proteasome 26S subunit, non-ATPase 7	PF3D7_1338100	MAL13P1.190	proteasome regulatory component, putative [Plasmodium falciparum 3D7]	Global	GO:0006511

PSMD8	proteasome 26S subunit, non-ATPase 8	PF3D7_1338100	MAL13P1.190	proteasome regulatory component, putative [Plasmodium falciparum 3D7]	Global	GO:0006511
PTBP1	polypyrimidine tract binding protein 1	PF3D7_0317200	PFC0755c	protein kinase, putative [Plasmodium falciparum 3D7]	Schizont	GO:0006468
PTBP1	polypyrimidine tract binding protein 1	PF3D7_1323400	PF13_0132	60S ribosomal protein L23a, putative [Plasmodium falciparum 3D7]	Ring	GO:0006412
PTBP1	polypyrimidine tract binding protein 1	PF3D7_1367100	MAL13P1.338	U1 small nuclear ribonucleoprotein, putative [Plasmodium falciparum 3D7]	Global	GO:0006396
PWP1	PWP1 homolog, endonuclein	PF3D7_0816000	PF08_0065	nucleolar preribosomal assembly protein, putative [Plasmodium falciparum 3D7]	Ring	None
RAB10	RAB10, member RAS oncogene family	PF3D7_0317200	PFC0755c	protein kinase, putative [Plasmodium falciparum 3D7]	Schizont	GO:0006468
RAB10	RAB10, member RAS oncogene family	PF3D7_0932800	PFI1590c	conserved Plasmodium protein, unknown function [Plasmodium falciparum 3D7]	Unknown	GO:0006886
RAB13	RAB13, member RAS oncogene family	PF3D7_0317200	PFC0755c	protein kinase, putative [Plasmodium falciparum 3D7]	Schizont	GO:0006468

RAB13	RAB13, member RAS oncogene family	PF3D7_0932 800	PFI1590c	conserved Plasmodium protein, unknown function [Plasmodium falciparum 3D7]	Unknown	GO:0006886
RAB14	RAB14, member RAS oncogene family	PF3D7_0317 200	PFC0755c	protein kinase, putative [Plasmodium falciparum 3D7]	Schizont	GO:0006468
RAB18	RAB18, member RAS oncogene family	PF3D7_0317 200	PFC0755c	protein kinase, putative [Plasmodium falciparum 3D7]	Schizont	GO:0006468
RAB18	RAB18, member RAS oncogene family	PF3D7_0932 800	PFI1590c	conserved Plasmodium protein, unknown function [Plasmodium falciparum 3D7]	Unknown	GO:0006886
RAB1A	RAB1A, member RAS oncogene family	PF3D7_0317 200	PFC0755c	protein kinase, putative [Plasmodium falciparum 3D7]	Schizont	GO:0006468
RAB1A	RAB1A, member RAS oncogene family	PF3D7_0932 800	PFI1590c	conserved Plasmodium protein, unknown function [Plasmodium falciparum 3D7]	Unknown	GO:0006886
RAB1B	RAB1B, member RAS oncogene family	PF3D7_0317 200	PFC0755c	protein kinase, putative [Plasmodium falciparum 3D7]	Schizont	GO:0006468
RAB1B	RAB1B, member RAS oncogene family	PF3D7_0932 800	PFI1590c	conserved Plasmodium protein, unknown function [Plasmodium falciparum 3D7]	Unknown	GO:0006886

RAB21	RAB21, member RAS oncogene family	PF3D7_0317200	PFC0755c	protein kinase, putative [Plasmodium falciparum 3D7]	Schizont	GO:0006468
RAB21	RAB21, member RAS oncogene family	PF3D7_0932800	PFI1590c	conserved Plasmodium protein, unknown function [Plasmodium falciparum 3D7]	Unknown	GO:0006886
RAB28	RAB28, member RAS oncogene family	PF3D7_0317200	PFC0755c	protein kinase, putative [Plasmodium falciparum 3D7]	Schizont	GO:0006468
RAB2A	RAB2A, member RAS oncogene family	PF3D7_0317200	PFC0755c	protein kinase, putative [Plasmodium falciparum 3D7]	Schizont	GO:0006468
RAB2B	RAB2B, member RAS oncogene family	PF3D7_0317200	PFC0755c	protein kinase, putative [Plasmodium falciparum 3D7]	Schizont	GO:0006468
RAB35	RAB35, member RAS oncogene family	PF3D7_0317200	PFC0755c	protein kinase, putative [Plasmodium falciparum 3D7]	Schizont	GO:0006468
RAB35	RAB35, member RAS oncogene family	PF3D7_0932800	PFI1590c	conserved Plasmodium protein, unknown function [Plasmodium falciparum 3D7]	Unknown	GO:0006886
RAB4A	RAB4A, member RAS oncogene family	PF3D7_0317200	PFC0755c	protein kinase, putative [Plasmodium falciparum 3D7]	Schizont	GO:0006468

RAB4A	RAB4A, member RAS oncogene family	PF3D7_0932 800	PFI1590c	conserved Plasmodium protein, unknown function [Plasmodium falciparum 3D7]	Unknown	GO:0006886
RAB4B	RAB4B, member RAS oncogene family	PF3D7_0317 200	PFC0755c	protein kinase, putative [Plasmodium falciparum 3D7]	Schizont	GO:0006468
RAB4B	RAB4B, member RAS oncogene family	PF3D7_0932 800	PFI1590c	conserved Plasmodium protein, unknown function [Plasmodium falciparum 3D7]	Unknown	GO:0006886
RAB5B	RAB5B, member RAS oncogene family	PF3D7_0317 200	PFC0755c	protein kinase, putative [Plasmodium falciparum 3D7]	Schizont	GO:0006468
RAB5B	RAB5B, member RAS oncogene family	PF3D7_0932 800	PFI1590c	conserved Plasmodium protein, unknown function [Plasmodium falciparum 3D7]	Unknown	GO:0006886
RAB5C	RAB5C, member RAS oncogene family	PF3D7_0317 200	PFC0755c	protein kinase, putative [Plasmodium falciparum 3D7]	Schizont	GO:0006468
RAB5C	RAB5C, member RAS oncogene family	PF3D7_0932 800	PFI1590c	conserved Plasmodium protein, unknown function [Plasmodium falciparum 3D7]	Unknown	GO:0006886
RAB6B	RAB6B, member RAS oncogene family	PF3D7_0317 200	PFC0755c	protein kinase, putative [Plasmodium falciparum 3D7]	Schizont	GO:0006468

RAB6C	RAB6C, member RAS oncogene family	PF3D7_0317 200	PFC0755c	protein kinase, putative [Plasmodium falciparum 3D7]	Schizont	GO:0006468
RAB7A	RAB7A, member RAS oncogene family	PF3D7_0317 200	PFC0755c	protein kinase, putative [Plasmodium falciparum 3D7]	Schizont	GO:0006468
RAB7A	RAB7A, member RAS oncogene family	PF3D7_0932 800	PFI1590c	conserved Plasmodium protein, unknown function [Plasmodium falciparum 3D7]	Unknown	GO:0006886
RAB8A	RAB8A, member RAS oncogene family	PF3D7_0317 200	PFC0755c	protein kinase, putative [Plasmodium falciparum 3D7]	Schizont	GO:0006468
RAB8A	RAB8A, member RAS oncogene family	PF3D7_0932 800	PFI1590c	conserved Plasmodium protein, unknown function [Plasmodium falciparum 3D7]	Unknown	GO:0006886
RAB8B	RAB8B, member RAS oncogene family	PF3D7_0317 200	PFC0755c	protein kinase, putative [Plasmodium falciparum 3D7]	Schizont	GO:0006468
RAB8B	RAB8B, member RAS oncogene family	PF3D7_0932 800	PFI1590c	conserved Plasmodium protein, unknown function [Plasmodium falciparum 3D7]	Unknown	GO:0006886
RAC1	Rac family small GTPase 1	PF3D7_0317 200	PFC0755c	protein kinase, putative [Plasmodium falciparum 3D7]	Schizont	GO:0006468

RALA	RAS like proto-oncogene A	PF3D7_0317200	PFC0755c	protein kinase, putative [Plasmodium falciparum 3D7]	Schizont	GO:0006468
RAN	RAN, member RAS oncogene family	PF3D7_0317200	PFC0755c	protein kinase, putative [Plasmodium falciparum 3D7]	Schizont	GO:0006468
RAN	RAN, member RAS oncogene family	PF3D7_0932800	PFI1590c	conserved Plasmodium protein, unknown function [Plasmodium falciparum 3D7]	Unknown	GO:0006886
RAP1A	RAP1A, member of RAS oncogene family	PF3D7_0317200	PFC0755c	protein kinase, putative [Plasmodium falciparum 3D7]	Schizont	GO:0006468
RAP1B	RAP1B, member of RAS oncogene family	PF3D7_0317200	PFC0755c	protein kinase, putative [Plasmodium falciparum 3D7]	Schizont	GO:0006468
RAP2A	RAP2A, member of RAS oncogene family	PF3D7_0317200	PFC0755c	protein kinase, putative [Plasmodium falciparum 3D7]	Schizont	GO:0006468
RAP2B	RAP2B, member of RAS oncogene family	PF3D7_0317200	PFC0755c	protein kinase, putative [Plasmodium falciparum 3D7]	Schizont	GO:0006468
RAP2C	RAP2C, member of RAS oncogene family	PF3D7_0317200	PFC0755c	protein kinase, putative [Plasmodium falciparum 3D7]	Schizont	GO:0006468
RASA2	RAS p21 protein activator 2	PF3D7_0317200	PFC0755c	protein kinase, putative [Plasmodium falciparum 3D7]	Schizont	GO:0006468

RASA2	RAS p21 protein activator 2	PF3D7_1121300	PF11_0220	hypothetical protein	Troph/ Gametocytes	
RASA2	RAS p21 protein activator 2	PF3D7_1243900	PFL2110c	conserved protein [Plasmodium falciparum 3D7]		
RASA2	RAS p21 protein activator 2	PF3D7_1359800	MAL13P1.297			
RASD1	ras related dexamethasone induced 1	PF3D7_0317200	PFC0755c	protein kinase, putative [Plasmodium falciparum 3D7]	Schizont	GO:0006468
RBBP7	RB binding protein 7, chromatin remodeling factor	PF3D7_0816000	PF08_0065	nucleolar preribosomal assembly protein, putative [Plasmodium falciparum 3D7]	Ring	None
RBM38	RNA binding motif protein 38	PF3D7_0317200	PFC0755c	protein kinase, putative [Plasmodium falciparum 3D7]	Schizont	GO:0006468
RBM38	RNA binding motif protein 38	PF3D7_1323400	PF13_0132	60S ribosomal protein L23a, putative [Plasmodium falciparum 3D7]	Ring	GO:0006412
RBM8A	RNA binding motif protein 8A	PF3D7_0317200	PFC0755c	protein kinase, putative [Plasmodium falciparum 3D7]	Schizont	GO:0006468
RBM8A	RNA binding motif protein 8A	PF3D7_1323400	PF13_0132	60S ribosomal protein L23a, putative [Plasmodium falciparum 3D7]	Ring	GO:0006412
RBM8A	RNA binding motif protein 8A	PF3D7_1367100	MAL13P1.338	U1 small nuclear ribonucleoprotein, putative [Plasmodium falciparum 3D7]	Global	GO:0006396

RBMX	RNA binding motif protein X-linked	PF3D7_0317 200	PFC0755c	protein kinase, putative [Plasmodium falciparum 3D7]	Schizont	GO:0006468
RBMX	RNA binding motif protein X-linked	PF3D7_1323 400	PF13_0132	60S ribosomal protein L23a, putative [Plasmodium falciparum 3D7]	Ring	GO:0006412
RBMX	RNA binding motif protein X-linked	PF3D7_1367 100	MAL13P1.338	U1 small nuclear ribonucleopr otein, putative [Plasmodium falciparum 3D7]	Global	GO:0006396
RGS10	regulator of G protein signaling 10	PF3D7_0317 200	PFC0755c	protein kinase, putative [Plasmodium falciparum 3D7]	Schizont	GO:0006468
RHOA	ras homolog family member A	PF3D7_0317 200	PFC0755c	protein kinase, putative [Plasmodium falciparum 3D7]	Schizont	GO:0006468
RHOB	ras homolog family member B	PF3D7_0317 200	PFC0755c	protein kinase, putative [Plasmodium falciparum 3D7]	Schizont	GO:0006468
RNH1	ribonuclease/ angiogenin inhibitor 1	PF3D7_0212 300	PF02_0115			
ROCK1	Rho associated coiled-coil containing protein kinase 1	PF3D7_0309 000	PFC0380w	dual-specificity protein phosphatase , putative	Troph/ Gametocytes	
ROCK1	Rho associated coiled-coil containing protein kinase 1	PF3D7_0317 200	PFC0755c	protein kinase, putative [Plasmodium falciparum 3D7]	Schizont	GO:0006468

ROCK1	Rho associated coiled-coil containing protein kinase 1	PF3D7_1030800	PF10_0301	calmodulin, putative [Plasmodium falciparum 3D7]	Global	GO:0005509
ROCK1	Rho associated coiled-coil containing protein kinase 1	PF3D7_1121300	PF11_0220	hypothetical protein	Troph/ Gametocytes	
ROCK1	Rho associated coiled-coil containing protein kinase 1	PF3D7_1135100	PF11_0362	protein phosphatase, putative [Plasmodium falciparum 3D7]	Ring/Schizont	None
ROCK1	Rho associated coiled-coil containing protein kinase 1	PF3D7_1247400	PFL2275c	FK506-binding protein (FKBP)-type peptidyl-propyl isomerase [Plasmodium falciparum 3D7]	Troph	GO:0006457
ROCK1	Rho associated coiled-coil containing protein kinase 1	PF3D7_1342400	PF13_0232	casein kinase II beta chain [Plasmodium falciparum 3D7]	Troph	GO:0004674
ROCK2	Rho associated coiled-coil containing protein kinase 2	PF3D7_0309000	PFC0380w	dual-specificity protein phosphatase, putative	Troph/ Gametocytes	
ROCK2	Rho associated coiled-coil containing protein kinase 2	PF3D7_0317200	PFC0755c	protein kinase, putative [Plasmodium falciparum 3D7]	Schizont	GO:0006468
ROCK2	Rho associated coiled-coil containing protein kinase 2	PF3D7_1030800	PF10_0301	calmodulin, putative [Plasmodium falciparum 3D7]	Global	GO:0005509
ROCK2	Rho associated coiled-coil containing protein kinase 2	PF3D7_1121300	PF11_0220	hypothetical protein	Troph/ Gametocytes	

ROCK2	Rho associated coiled-coil containing protein kinase 2	PF3D7_1135100	PF11_0362	protein phosphatase, putative [Plasmodium falciparum 3D7]	Ring/Schizont	None
ROCK2	Rho associated coiled-coil containing protein kinase 2	PF3D7_1247400	PFL2275c	FK506-binding protein (FKBP)-type peptidyl-propyl isomerase [Plasmodium falciparum 3D7]	Troph	GO:0006457
ROCK2	Rho associated coiled-coil containing protein kinase 2	PF3D7_1342400	PF13_0232	casein kinase II beta chain [Plasmodium falciparum 3D7]	Troph	GO:0004674
RPIA	ribose 5-phosphate isomerase A	PF3D7_0514600	PFE0730c	ribose 5-phosphate epimerase, putative	Merozoites	
RPTOR	regulatory associated protein of MTOR complex 1	PF3D7_0317200	PFC0755c	protein kinase, putative [Plasmodium falciparum 3D7]	Schizont	GO:0006468
RPTOR	regulatory associated protein of MTOR complex 1	PF3D7_0816000	PF08_0065	nucleolar preribosomal assembly protein, putative [Plasmodium falciparum 3D7]	Ring	None
RPTOR	regulatory associated protein of MTOR complex 1	PF3D7_1036700	PF10_0359			
RPTOR	regulatory associated protein of MTOR complex 1	PF3D7_1329300	PF13_0149	chromatin assembly factor 1 subunit, putative [Plasmodium falciparum 3D7]	Global	GO:0006333
RSU1	Ras suppressor protein 1	PF3D7_0616500	PFF0800w			

RWDD1	RWD domain containing 1	PF3D7_1358900	MAL13P1.294	GTP binding protein, putative [Plasmodium falciparum 3D7]	Global	GO:0007165
SAR1B	secretion associated Ras related GTPase 1B	PF3D7_1359800	MAL13P1.297			
SAR1B	secretion associated Ras related GTPase 1B	PF3D7_1442900	PF14_0407	guanine nucleotide exchange factor, putative [Plasmodium falciparum 3D7]	Ring	GO:0032012
SBDS	SBDS ribosome maturation factor	PF3D7_0602400	PFF0115c			
SCFD1	sec1 family domain containing 1	PF3D7_0613700	PFF0665c	syntaxin binding protein, putative [Plasmodium falciparum 3D7]	Global	GO:0006904
SCYL1	SCY1 like pseudokinase 1	PF3D7_0309000	PFC0380w	dual-specificity protein phosphatase, putative	Troph/ Gametocytes	
SCYL1	SCY1 like pseudokinase 1	PF3D7_0317200	PFC0755c	protein kinase, putative [Plasmodium falciparum 3D7]	Schizont	GO:0006468
SCYL1	SCY1 like pseudokinase 1	PF3D7_1121300	PF11_0220	hypothetical protein	Troph/ Gametocytes	
SCYL1	SCY1 like pseudokinase 1	PF3D7_1135100	PF11_0362	protein phosphatase, putative [Plasmodium falciparum 3D7]	Ring/Schizont	None

SCYL1	SCY1 like pseudokinase 1	PF3D7_1247400	PFL2275c	FK506-binding protein (FKBP)-type peptidyl-propyl isomerase [Plasmodium falciparum 3D7]	Troph	GO:0006457
SCYL1	SCY1 like pseudokinase 1	PF3D7_1342400	PF13_0232	casein kinase II beta chain [Plasmodium falciparum 3D7]	Troph	GO:0004674
SEC13	SEC13 homolog, nuclear pore and COPII coat complex component	PF3D7_1036700	PF10_0359			
SEC13	SEC13 homolog, nuclear pore and COPII coat complex component	PF3D7_1122900	PF11_0240	dynein heavy chain, putative	Merozoites/ Gametocytes	
SEC13	SEC13 homolog, nuclear pore and COPII coat complex component	PF3D7_1329300	PF13_0149	chromatin assembly factor 1 subunit, putative [Plasmodium falciparum 3D7]	Global	GO:0006333
SF3B4	splicing factor 3b subunit 4	PF3D7_0317200	PFC0755c	protein kinase, putative [Plasmodium falciparum 3D7]	Schizont	GO:0006468
SF3B4	splicing factor 3b subunit 4	PF3D7_1323400	PF13_0132	60S ribosomal protein L23a, putative [Plasmodium falciparum 3D7]	Ring	GO:0006412
SF3B4	splicing factor 3b subunit 4	PF3D7_1367100	MAL13P1.338	U1 small nuclear ribonucleoprotein, putative [Plasmodium falciparum 3D7]	Global	GO:0006396

SHC1	SHC adaptor protein 1	PF3D7_0515 300	PFE0765w	phosphatidylinositol 3-kinase, putative [Plasmodium falciparum 3D7]	Global	GO:0006897
SHC1	SHC adaptor protein 1	PF3D7_1121 300	PF11_0220	hypothetical protein	Troph/ Gametocytes	
SHC1	SHC adaptor protein 1	PF3D7_1243 900	PFL2110c	conserved protein [Plasmodium falciparum 3D7]		
SHPK	sedoheptulokinase	PF3D7_1351 600	PF13_0269			
SLK	STE20 like kinase	PF3D7_0309 000	PFC0380w	dual-specificity protein phosphatase, putative	Troph/ Gametocytes	
SLK	STE20 like kinase	PF3D7_0317 200	PFC0755c	protein kinase, putative [Plasmodium falciparum 3D7]	Schizont	GO:0006468
SLK	STE20 like kinase	PF3D7_1030 800	PF10_0301	calmodulin, putative [Plasmodium falciparum 3D7]	Global	GO:0005509
SLK	STE20 like kinase	PF3D7_1121 300	PF11_0220	hypothetical protein	Troph/ Gametocytes	
SLK	STE20 like kinase	PF3D7_1135 100	PF11_0362	protein phosphatase, putative [Plasmodium falciparum 3D7]	Ring/Schizont	None
SLK	STE20 like kinase	PF3D7_1247 400	PFL2275c	FK506-binding protein (FKBP)-type peptidyl-propyl isomerase [Plasmodium falciparum 3D7]	Troph	GO:0006457
SLK	STE20 like kinase	PF3D7_1342 400	PF13_0232	casein kinase II beta chain [Plasmodium falciparum 3D7]	Troph	GO:0004674
SMAP2	small ArfGAP2	PF3D7_1359 800	MAL13P1.297			

SNX9	sorting nexin 9	PF3D7_1121300	PF11_0220	hypothetical protein	Troph/ Gametocytes	
SPTA1	spectrin alpha, erythrocytic 1	PF3D7_0613900	PFF0675c	myosin E [Plasmodium falciparum 3D7]	Schizont	GO:0003774
SPTA1	spectrin alpha, erythrocytic 1	PF3D7_1121300	PF11_0220	hypothetical protein	Troph/ Gametocytes	
SRPK1	SRSF protein kinase 1	PF3D7_0309000	PFC0380w	dual-specificity protein phosphatase, putative	Troph/ Gametocytes	
SRPK1	SRSF protein kinase 1	PF3D7_0317200	PFC0755c	protein kinase, putative [Plasmodium falciparum 3D7]	Schizont	GO:0006468
SRPK1	SRSF protein kinase 1	PF3D7_1121300	PF11_0220	hypothetical protein	Troph/ Gametocytes	
SRPK1	SRSF protein kinase 1	PF3D7_1135100	PF11_0362	protein phosphatase, putative [Plasmodium falciparum 3D7]	Ring/Schizont	None
SRPK1	SRSF protein kinase 1	PF3D7_1247400	PFL2275c	FK506-binding protein (FKBP)-type peptidyl-propyl isomerase [Plasmodium falciparum 3D7]	Troph	GO:0006457
SRPK1	SRSF protein kinase 1	PF3D7_1342400	PF13_0232	casein kinase II beta chain [Plasmodium falciparum 3D7]	Troph	GO:0004674
STAT6	signal transducer and activator of transcription 6	PF3D7_1121300	PF11_0220	hypothetical protein	Troph/ Gametocytes	
STAT6	signal transducer and activator of transcription 6	PF3D7_1243900	PFL2110c	conserved protein [Plasmodium falciparum 3D7]		

STIP1	stress induced phosphoprotein 1	PF3D7_0515300	PFE0765w	phosphatidylinositol 3-kinase, putative [Plasmodium falciparum 3D7]	Global	GO:0006897
STIP1	stress induced phosphoprotein 1	PF3D7_0601600	PFF0080c			
STIP1	stress induced phosphoprotein 1	PF3D7_0631000	PFF1505w	TPR-like domain containing protein, putative [Plasmodium falciparum 3D7]		
STK10	serine/threonine kinase 10	PF3D7_0309000	PFC0380w	dual-specificity protein phosphatase, putative	Troph/Gametocytes	
STK10	serine/threonine kinase 10	PF3D7_0317200	PFC0755c	protein kinase, putative [Plasmodium falciparum 3D7]	Schizont	GO:0006468
STK10	serine/threonine kinase 10	PF3D7_1030800	PF10_0301	calmodulin, putative [Plasmodium falciparum 3D7]	Global	GO:0005509
STK10	serine/threonine kinase 10	PF3D7_1121300	PF11_0220	hypothetical protein	Troph/Gametocytes	
STK10	serine/threonine kinase 10	PF3D7_1135100	PF11_0362	protein phosphatase, putative [Plasmodium falciparum 3D7]	Ring/Schizont	None
STK10	serine/threonine kinase 10	PF3D7_1247400	PFL2275c	FK506-binding protein (FKBP)-type peptidyl-propyl isomerase [Plasmodium falciparum 3D7]	Troph	GO:0006457

STK10	serine/ threonine kinase 10	PF3D7_1342 400	PF13_0232	casein kinase II beta chain [Plasmodium falciparum 3D7]	Troph	GO:0004674
STK24	serine/ threonine kinase 24	PF3D7_0309 000	PFC0380w	dual-specificity protein phosphatase , putative	Troph/ Gametocytes	
STK24	serine/ threonine kinase 24	PF3D7_0317 200	PFC0755c	protein kinase, putative [Plasmodium falciparum 3D7]	Schizont	GO:0006468
STK24	serine/ threonine kinase 24	PF3D7_1030 800	PF10_0301	calmodulin, putative [Plasmodium falciparum 3D7]	Global	GO:0005509
STK24	serine/ threonine kinase 24	PF3D7_1121 300	PF11_0220	hypothetical protein	Troph/ Gametocytes	
STK24	serine/ threonine kinase 24	PF3D7_1135 100	PF11_0362	protein phosphatase , putative [Plasmodium falciparum 3D7]	Ring/Schizont	None
STK24	serine/ threonine kinase 24	PF3D7_1247 400	PFL2275c	FK506-binding protein (FKBP)-type peptidyl-propyl isomerase [Plasmodium falciparum 3D7]	Troph	GO:0006457
STK24	serine/ threonine kinase 24	PF3D7_1342 400	PF13_0232	casein kinase II beta chain [Plasmodium falciparum 3D7]	Troph	GO:0004674
STK38	serine/ threonine kinase 38	PF3D7_0309 000	PFC0380w	dual-specificity protein phosphatase , putative	Troph/ Gametocytes	
STK38	serine/ threonine kinase 38	PF3D7_0317 200	PFC0755c	protein kinase, putative [Plasmodium falciparum 3D7]	Schizont	GO:0006468

STK38	serine/ threonine kinase 38	PF3D7_1030800	PF10_0301	calmodulin, putative [Plasmodium falciparum 3D7]	Global	GO:0005509
STK38	serine/ threonine kinase 38	PF3D7_1121300	PF11_0220	hypothetical protein	Troph/ Gametocytes	
STK38	serine/ threonine kinase 38	PF3D7_1135100	PF11_0362	protein phosphatase, putative [Plasmodium falciparum 3D7]	Ring/Schizont	None
STK38	serine/ threonine kinase 38	PF3D7_1247400	PFL2275c	FK506-binding protein (FKBP)-type peptidyl-propyl isomerase [Plasmodium falciparum 3D7]	Troph	GO:0006457
STK38	serine/ threonine kinase 38	PF3D7_1342400	PF13_0232	casein kinase II beta chain [Plasmodium falciparum 3D7]	Troph	GO:0004674
STRAP	serine/ threonine kinase receptor associated protein	PF3D7_1036700	PF10_0359			
STX16	syntaxin 16	PF3D7_0613700	PFF0665c	syntaxin binding protein, putative [Plasmodium falciparum 3D7]	Global	GO:0006904
STX16	syntaxin 16	PF3D7_1243900	PFL2110c	conserved protein [Plasmodium falciparum 3D7]		
STX4	syntaxin 4	PF3D7_0613700	PFF0665c	syntaxin binding protein, putative [Plasmodium falciparum 3D7]	Global	GO:0006904

STX4	syntaxin 4	PF3D7_1243900	PFL2110c	conserved protein [Plasmodium falciparum 3D7]		
STX6	syntaxin 6	PF3D7_0613700	PFF0665c	syntaxin binding protein, putative [Plasmodium falciparum 3D7]	Global	GO:0006904
STX6	syntaxin 6	PF3D7_1243900	PFL2110c	conserved protein [Plasmodium falciparum 3D7]		
STX7	syntaxin 7	PF3D7_0613700	PFF0665c	syntaxin binding protein, putative [Plasmodium falciparum 3D7]	Global	GO:0006904
STX7	syntaxin 7	PF3D7_1243900	PFL2110c	conserved protein [Plasmodium falciparum 3D7]		
SYK	spleen associated tyrosine kinase	PF3D7_0514300	PFE0715w	aspartyl t-RNA synthetase, putative	Merozoites	
SYK	spleen associated tyrosine kinase	PF3D7_0628800	PFF1395c			
TAOK3	TAO kinase 3	PF3D7_0309000	PFC0380w	dual-specificity protein phosphatase, putative	Troph/ Gametocytes	
TAOK3	TAO kinase 3	PF3D7_0317200	PFC0755c	protein kinase, putative [Plasmodium falciparum 3D7]	Schizont	GO:0006468
TAOK3	TAO kinase 3	PF3D7_1030800	PF10_0301	calmodulin, putative [Plasmodium falciparum 3D7]	Global	GO:0005509
TAOK3	TAO kinase 3	PF3D7_1121300	PF11_0220	hypothetical protein	Troph/ Gametocytes	

TAOK3	TAO kinase 3	PF3D7_1135100	PF11_0362	protein phosphatase, putative [Plasmodium falciparum 3D7]	Ring/Schizont	None
TAOK3	TAO kinase 3	PF3D7_1247400	PFL2275c	FK506-binding protein (FKBP)-type peptidyl-propyl isomerase [Plasmodium falciparum 3D7]	Troph	GO:0006457
TAOK3	TAO kinase 3	PF3D7_1342400	PF13_0232	casein kinase II beta chain [Plasmodium falciparum 3D7]	Troph	GO:0004674
TBCB	tubulin folding cofactor B	PF3D7_0906910	PFI0335w	tubulin-specific chaperone, putative [Plasmodium falciparum 3D7]	Global	None
TCEA1	transcription elongation factor A1	PF3D7_0215700	PF02_0148			
TFG	trafficking from ER to golgi regulator	PF3D7_0317200	PFC0755c	protein kinase, putative [Plasmodium falciparum 3D7]	Schizont	GO:0006468
TKT	transketolase	PF3D7_0820700	PF08_0045	2-oxoglutarate dehydrogenase E1 component [Plasmodium falciparum 3D7]	Global	GO:0006096
TLN1	talin 1	PF3D7_1121300	PF11_0220	hypothetical protein	Troph/ Gametocytes	
TLN2	talin 2	PF3D7_1121300	PF11_0220	hypothetical protein	Troph/ Gametocytes	
TMX1	thioredoxin related transmembrane protein 1	PF3D7_1411400	PF14_0112			

TNPO1	transportin 1	PF3D7_0932800	PFI1590c	conserved Plasmodium protein, unknown function [Plasmodium falciparum 3D7]	Unknown	GO:0006886
TNPO2	transportin 2	PF3D7_0932800	PFI1590c	conserved Plasmodium protein, unknown function [Plasmodium falciparum 3D7]	Unknown	GO:0006886
TRAP1	TNF receptor associated protein 1	PF3D7_0317200	PFC0755c	protein kinase, putative [Plasmodium falciparum 3D7]	Schizont	GO:0006468
TRAP1	TNF receptor associated protein 1	PF3D7_1239500	PFL1915w			
TWF1	twinfilin actin binding protein 1	PF3D7_0823300	PF08_0034	histone acetyltransferase GCN5, putative [Plasmodium falciparum 3D7]	Global	GO:0006355
TWF1	twinfilin actin binding protein 1	PF3D7_1121300	PF11_0220	hypothetical protein	Troph/ Gametocytes	
TWF2	twinfilin actin binding protein 2	PF3D7_0823300	PF08_0034	histone acetyltransferase GCN5, putative [Plasmodium falciparum 3D7]	Global	GO:0006355
TWF2	twinfilin actin binding protein 2	PF3D7_1121300	PF11_0220	hypothetical protein	Troph/ Gametocytes	
TXNL1	thioredoxin like 1	PF3D7_1411400	PF14_0112			
UBA3	ubiquitin like modifier activating enzyme 3	PF3D7_1237000	PFL1790w	ubiquitin-activating enzyme, putative [Plasmodium falciparum 3D7]	Global	GO:0006464

UBA6	ubiquitin like modifier activating enzyme 6	PF3D7_1237000	PFL1790w	ubiquitin-activating enzyme, putative [Plasmodium falciparum 3D7]	Global	GO:0006464
UBAC1	UBA domain containing 1	PF3D7_0317200	PFC0755c	protein kinase, putative [Plasmodium falciparum 3D7]	Schizont	GO:0006468
UBC	ubiquitin C	PF3D7_1355700	MAL13P1.275	protein phosphatase, putative [Plasmodium falciparum 3D7]	Global	None
UBE2A	ubiquitin conjugating enzyme E2 A	PF3D7_1237000	PFL1790w	ubiquitin-activating enzyme, putative [Plasmodium falciparum 3D7]	Global	GO:0006464
UBE2A	ubiquitin conjugating enzyme E2 A	PF3D7_1361900	PF13_0328	proliferating cell nuclear antigen [Plasmodium falciparum 3D7]	Troph	GO:0006275
UBE2H	ubiquitin conjugating enzyme E2 H	PF3D7_1237000	PFL1790w	ubiquitin-activating enzyme, putative [Plasmodium falciparum 3D7]	Global	GO:0006464
UBE2H	ubiquitin conjugating enzyme E2 H	PF3D7_1361900	PF13_0328	proliferating cell nuclear antigen [Plasmodium falciparum 3D7]	Troph	GO:0006275
UBE2K	ubiquitin conjugating enzyme E2 K	PF3D7_0317200	PFC0755c	protein kinase, putative [Plasmodium falciparum 3D7]	Schizont	GO:0006468
UBE2K	ubiquitin conjugating enzyme E2 K	PF3D7_1237000	PFL1790w	ubiquitin-activating enzyme, putative [Plasmodium falciparum 3D7]	Global	GO:0006464

UBE2K	ubiquitin conjugating enzyme E2 K	PF3D7_1361900	PF13_0328	proliferating cell nuclear antigen [Plasmodium falciparum 3D7]	Troph	GO:0006275
UBE2N	ubiquitin conjugating enzyme E2 N	PF3D7_1237000	PFL1790w	ubiquitin-activating enzyme, putative [Plasmodium falciparum 3D7]	Global	GO:0006464
UBE2N	ubiquitin conjugating enzyme E2 N	PF3D7_1361900	PF13_0328	proliferating cell nuclear antigen [Plasmodium falciparum 3D7]	Troph	GO:0006275
UBE2O	ubiquitin conjugating enzyme E2 O	PF3D7_1237000	PFL1790w	ubiquitin-activating enzyme, putative [Plasmodium falciparum 3D7]	Global	GO:0006464
UBE2O	ubiquitin conjugating enzyme E2 O	PF3D7_1361900	PF13_0328	proliferating cell nuclear antigen [Plasmodium falciparum 3D7]	Troph	GO:0006275
UBE3B	ubiquitin protein ligase E3B	PF3D7_0613900	PFF0675c	myosin E [Plasmodium falciparum 3D7]	Schizont	GO:0003774
UBE3B	ubiquitin protein ligase E3B	PF3D7_1030800	PF10_0301	calmodulin, putative [Plasmodium falciparum 3D7]	Global	GO:0005509
UBL4A	ubiquitin like 4A	PF3D7_0317200	PFC0755c	protein kinase, putative [Plasmodium falciparum 3D7]	Schizont	GO:0006468
UBL4A	ubiquitin like 4A	PF3D7_1355700	MAL13P1.275	protein phosphatase, putative [Plasmodium falciparum 3D7]	Global	None

UBL4A	ubiquitin like 4A	PF3D7_1361900	PF13_0328	proliferating cell nuclear antigen [Plasmodium falciparum 3D7]	Troph	GO:0006275
UBL7	ubiquitin like 7	PF3D7_0317200	PFC0755c	protein kinase, putative [Plasmodium falciparum 3D7]	Schizont	GO:0006468
UBXN1	UBX domain protein 1	PF3D7_0317200	PFC0755c	protein kinase, putative [Plasmodium falciparum 3D7]	Schizont	GO:0006468
ULK3	unc-51 like kinase 3	PF3D7_0309000	PFC0380w	dual-specificity protein phosphatase, putative	Troph/ Gametocytes	
ULK3	unc-51 like kinase 3	PF3D7_0317200	PFC0755c	protein kinase, putative [Plasmodium falciparum 3D7]	Schizont	GO:0006468
ULK3	unc-51 like kinase 3	PF3D7_1030800	PF10_0301	calmodulin, putative [Plasmodium falciparum 3D7]	Global	GO:0005509
ULK3	unc-51 like kinase 3	PF3D7_1121300	PF11_0220	hypothetical protein	Troph/ Gametocytes	
ULK3	unc-51 like kinase 3	PF3D7_1135100	PF11_0362	protein phosphatase, putative [Plasmodium falciparum 3D7]	Ring/Schizont	None
ULK3	unc-51 like kinase 3	PF3D7_1247400	PFL2275c	FK506-binding protein (FKBP)-type peptidyl-propyl isomerase [Plasmodium falciparum 3D7]	Troph	GO:0006457
ULK3	unc-51 like kinase 3	PF3D7_1342400	PF13_0232	casein kinase II beta chain [Plasmodium falciparum 3D7]	Troph	GO:0004674

USP9X	ubiquitin specific peptidase 9 X-linked	PF3D7_1329300	PF13_0149	chromatin assembly factor 1 subunit, putative [Plasmodium falciparum 3D7]	Global	GO:0006333
VPS45	vacuolar protein sorting 45 homolog	PF3D7_0613700	PFF0665c	syntaxin binding protein, putative [Plasmodium falciparum 3D7]	Global	GO:0006904
VPS4A	vacuolar protein sorting 4 homolog A	PF3D7_0816600	PF08_0063	ClpB protein, putative [Plasmodium falciparum 3D7]	Troph	GO:0000166
VPS4A	vacuolar protein sorting 4 homolog A	PF3D7_1116800	PF11_0175	heat shock protein 101, putative [Plasmodium falciparum 3D7]	Ring	GO:0006457
VPS4A	vacuolar protein sorting 4 homolog A	PF3D7_1361900	PF13_0328	proliferating cell nuclear antigen [Plasmodium falciparum 3D7]	Troph	GO:0006275
VPS4A	vacuolar protein sorting 4 homolog A	PF3D7_1406600	PF14_0063	ATP-dependent CLP protease, putative [Plasmodium falciparum 3D7]	Global	GO:0008462
VPS4B	vacuolar protein sorting 4 homolog B	PF3D7_0816600	PF08_0063	ClpB protein, putative [Plasmodium falciparum 3D7]	Troph	GO:0000166
VPS4B	vacuolar protein sorting 4 homolog B	PF3D7_1116800	PF11_0175	heat shock protein 101, putative [Plasmodium falciparum 3D7]	Ring	GO:0006457
VPS4B	vacuolar protein sorting 4 homolog B	PF3D7_1361900	PF13_0328	proliferating cell nuclear antigen [Plasmodium falciparum 3D7]	Troph	GO:0006275

WBP4	WW domain binding protein 4	PF3D7_1367100	MAL13P1.338	U1 small nuclear ribonucleoprotein, putative [Plasmodium falciparum 3D7]	Global	GO:0006396
WDFY1	WD repeat and FYVE domain containing 1	PF3D7_1036700	PF10_0359			
WDFY1	WD repeat and FYVE domain containing 1	PF3D7_1329300	PF13_0149	chromatin assembly factor 1 subunit, putative [Plasmodium falciparum 3D7]	Global	GO:0006333
WDR1	WD repeat domain 1	PF3D7_0909900	PFI0480w	helicase with Zn-finger motif, putative [Plasmodium falciparum 3D7]	Global	GO:0003676
WDR1	WD repeat domain 1	PF3D7_1036700	PF10_0359			
WDR1	WD repeat domain 1	PF3D7_1329300	PF13_0149	chromatin assembly factor 1 subunit, putative [Plasmodium falciparum 3D7]	Global	GO:0006333
WDR26	WD repeat domain 26	PF3D7_0816000	PF08_0065	nucleolar preribosomal assembly protein, putative [Plasmodium falciparum 3D7]	Ring	None
WDR44	WD repeat domain 44	PF3D7_0816000	PF08_0065	nucleolar preribosomal assembly protein, putative [Plasmodium falciparum 3D7]	Ring	None
WDR44	WD repeat domain 44	PF3D7_1036700	PF10_0359			

WDR44	WD repeat domain 44	PF3D7_1329300	PF13_0149	chromatin assembly factor 1 subunit, putative [Plasmodium falciparum 3D7]	Global	GO:0006333
WDR61	WD repeat domain 61	PF3D7_0816000	PF08_0065	nucleolar preribosomal assembly protein, putative [Plasmodium falciparum 3D7]	Ring	None
WDR61	WD repeat domain 61	PF3D7_0909900	PFI0480w	helicase with Zn-finger motif, putative [Plasmodium falciparum 3D7]	Global	GO:0003676
WNK1	WNK lysine deficient protein kinase 1	PF3D7_0309000	PFC0380w	dual-specificity protein phosphatase, putative	Troph/ Gametocytes	
WNK1	WNK lysine deficient protein kinase 1	PF3D7_0317200	PFC0755c	protein kinase, putative [Plasmodium falciparum 3D7]	Schizont	GO:0006468
WNK1	WNK lysine deficient protein kinase 1	PF3D7_1121300	PF11_0220	hypothetical protein	Troph/ Gametocytes	
WNK1	WNK lysine deficient protein kinase 1	PF3D7_1247400	PFL2275c	FK506-binding protein (FKBP)-type peptidyl-propyl isomerase [Plasmodium falciparum 3D7]	Troph	GO:0006457
WNK1	WNK lysine deficient protein kinase 1	PF3D7_1342400	PF13_0232	casein kinase II beta chain [Plasmodium falciparum 3D7]	Troph	GO:0004674

YKT6	YKT6 v-SNARE homolog	PF3D7_0405100	PFD0250c	Sec24-like protein, putative [Plasmodium falciparum 3D7]	Global	GO:0006886
